# Supplementary material for: Extreme genomic volatility characterizes the evolution of the immunoglobulin heavy chain locus in cyprinodontiform fishes
Source: Proc Biol Sci. 2020 May 13;287(1927):20200489. doi: 10.1098/rspb.2020.0489 (PMC7287348; doi:10.1098/rspb.2020.0489)
Supplement: Supplementary information [file rspb20200489supp1.pdf]

Supplementary Information

A Supplementary methods **2**

A.1 BAC insert assembly . . . 2

A.2 Collating reference sequences . . . 2

A.3 Identifying putative locus sequences . . . 3

A.4 Characterising gene segments . . . 4

A.5 Cross-sublocus sequence comparison . . . 7

A.6 Phylogenetic trees . . . 7

A.7 Data and code availability . . . 8

B Supplementary figures **9**

C Supplementary tables **21**

A Supplementary methods

A.1 BAC insert assembly

Demultiplexed, adapter-trimmed MiSeq sequencing data were uploaded by the sequencing provider to Illumina BaseSpace and accessed via the Illumina utility program BaseMount. Reads from each library were processed with Trimmomatic[40] to remove adapter sequences, trim low-quality sequence regions, and discard any trimmed reads below a minimum length:

```
trimmomatic PE -phred33 <forward_reads_fastq> <reverse_reads_fastq>
  ↪ <output_paths> ILLUMINACLIP:<adapter_directory>/TruSeq3-PE.fa:2:30:10
  ↪ LEADING:20 TRAILING:20 SLIDINGWINDOW:4:30 MINLEN:36
```

Following this, the trimmed reads were filtered to remove *E. coli* genomic DNA and other contaminants by aligning them using Bowtie 2[41] and retaining read pairs that did not align concordantly:

```
bowtie2 --very-sensitive-local --local --reorder --un-conc <output_prefix> -x
  ↪ <ecoli_genome_index_path> -1 <forward_reads_fastq> -2
  ↪ <reverse_reads_fastq> -S <sam_file_prefix>
```

Before sequence assembly, the filtered reads then underwent correction, to reduce the impact of errors occurring during the library preparation and sequencing process. In order to increase the reliability of the resulting scaffolds and reduce the impact of ideosyncracies of any given correction tool, the reads were corrected in parallel using two different programs; QuorUM[42]:

```
quorum -d -q "33" -p <output_path> <interleaved_reads_files>
```

and BayesHammer (the built-in correction tool of the SPAdes genome-assembly software[43,44]):

```
spades.py -1 <forward_reads_fastq> -2 <reverse_reads_fastq> -o <output_path>
  ↪ --disable-gzip-output --only-error-correction --careful --cov-cutoff
  ↪ auto -k 21,33,55,77,99,127 --phred-offset 33
```

Following read correction, each pair of independently-corrected reads files was passed to SPAdes[43] for *de novo* genome assembly:

```
spades.py -1 <forward_reads_fastq> -2 <reverse_reads_fastq> -o <output_path>
  ↪ --disable-gzip-output --only-assembler --careful --cov-cutoff auto -k
  ↪ 21,33,55,77,99,127 --phred-offset 33
```

Following assembly, any *E. coli* scaffolds resulting from residual contaminating reads were identified by aligning scaffolds to the *E. coli* genome using BLAST:

```
blastn -task megablast -best_hit_overhang 0.1 -best_hit_score_edge 0.1 -query
  ↪ <bac_scaffolds> -subject <ecoli_genome>
```

Scaffolds containing significant matches to *E. coli* were discarded. The remaining scaffolds were then scaffolded using SSPACE[45], using jumping libraries from the previously published killifish genome assemblies[16,17]:

```
SSPACE_Standard_v3.0.pl -x 0 -k 5 -a 0.7 -n 15 -z 200 -g 1 -p 0 -l
  ↪ <jumping_library_config_file> -s <spades_scaffolds_file>
```

The assemblies produced with BayesHammer- and QuorUM-corrected reads were then compared, broken into consensus segments, and reassembled through end-to-end PCR and sequencing, as described in the main text.

A.2 Collating reference sequences

Most publications presenting characterisations of *IGH* loci do not provide easy-to-use databases of trimmed and curated gene segments, and the data that is available is often partial and heterogeneous between publications. In order to obtain standardised reference databases for locus characterisation, further analysis was performed on publically-available data from three reference species with previously-characterised *IGH* loci: medaka (*Oryzias latipes*)[10], zebrafish (*Danio rerio*)[9] and three-spined stickleback (*Gasterosteus aculeatus*)[11,12].

A.2.1 Medaka

GenBank files of the annotated medaka *IGH* locus were downloaded from the supplementary information of the medaka locus paper[10] and corrected to make them parsable by the `genbankr` R package. Locus sequence and annotation ranges were extracted from these GenBank files into FASTA and tab-separated tabular formats, respectively, and segment annotations were renamed to match the naming conventions used in other species. VH, DH, JH and constant-region-exon nucleotide sequences were extracted from the locus sequence using these annotations. Amino-acid sequences for VH, JH and constant-region sequences were obtained automatically by identifying the reading frames which minimised the number of stop codons in each sequence.

A.2.2 Stickleback

Limited sequence information on the *IGH* locus in stickleback, including VH segments and bulk (non-exon-separated) constant regions was provided in a GenBank file in the locus characterisation paper for medaka[10], while additional sequence information (including DH and JH nucleic-acid sequences and amino-acid sequences of constant-region exons) was extracted manually from one of the stickleback locus paper[11] into FASTA files. As with medaka, the GenBank reference file was downloaded, corrected and parsed to yield a FASTA file of the locus sequence and tab-separated tabular files of annotation ranges. VH sequences were extracted from the locus sequence using these annotation ranges and translated as specified for medaka above; JH sequences provided by Bao *et al.*[11] were translated such that the final nucleotide formed the last position of the final codon.

To obtain nucleic-acid sequences of the constant-region exons, the amino-acid sequences from Bao *et al.*[11] were aligned to the locus sequence with TBLASTN[62], with a query coverage threshold of 40 % and a maximum of three HSPs per query sequence:

```
tblastn -query <ch_aa_fasta> -subject <gac_locus_fasta> -qcov_hsp_perc 40
    ↪ -max_hsps 3 -outfmt '<output_format>' > <output_path>
```

with the following standardised tabular output format:

```
6 qseqid sseqid pident qcovhsp length mismatch gapopen gaps sstrand qstart
    ↪ qend sstart send evalue bitscore qlen slen
```

To filter out alignments across subloci, any alignment of an exon upstream of the annotated boundaries of its corresponding bulk constant region (whose ranges were specified in the GenBank file) was discarded; the alignment with the highest score for each exon was then used to extract the corresponding nucleic-acid sequence from the locus. In order to control for any errors, either during manual copying of locus sequences from the source paper or in the paper itself, these nucleic-acid sequences were then re-translated to generate new amino-acid sequences, again using the translation frame producing the fewest stop codons.

A.2.3 Zebrafish

GenBank files corresponding to the zebrafish *IGH* locus were provided (without segment annotations) on GenBank by Danilova *et al.*[9]; this publication also provided detailed co-ordinates for the VH, DH and JH segments (but not constant exons) on these sequences. Aligned amino-acid sequences were provided for the exons of *IGHM* and *IGHZ*, but no detailed information about *IGHD* exons could be found; as a result, reference information about *IGHD* was not used from this species.

As with stickleback, the amino-acid sequences provided were aligned to the locus sequences with TBLASTN (using the same parameters as for stickleback) to identify and extract exon nucleic-acid sequences, which were then translated using the frame yielding the fewest stop codons for each sequence. VH sequences were obtained using the ranges provided in Danilova *et al.*[9] and translated in the same manner. DH and JH nucleotide sequences were obtained directly from Danilova *et al.*[9]; as with stickleback, JH amino-acid sequences were obtained by translating the nucleotide sequences in the frame such that the final nucleotide formed the last position of the final codon.

A.3 Identifying putative locus sequences

In order to identify sequences in a genome assembly potentially containing part of an *IGH* locus, reference VH, JH and constant-region nucleotide and amino-acid sequences were mapped to the assembly using BLAST[39]. Nucleotide sequences were aligned to the locus using the relatively permissive `blastn` algorithm:

```
blastn -task blastn -query <reference_exon_fasta> -subject <locus_fasta>
    ↪ -outfmt '<output_format>'
```

Protein sequences, meanwhile, were aligned using the standard `blastp` algorithm:

```
blastp -query <reference_exon_fasta> -subject <locus_fasta> -outfmt
    ↪ '<output_format>'
```

In both cases, the tabular output format specified in Section A.2 was used, to provide a predictable format for downstream processing of BLAST alignment tables.

Following alignment of reference sequences, overlapping alignments to reference segments of the same segment type, isotype (if applicable) and exon number (if applicable) were collapsed together, keeping track of the number of collapsed alignments and the best E-values and bitscores obtained for each alignment group. Alignment groups with a very poor maximum E-value (> 0.001) were discarded, as were groups consisting of fewer than two alignments and groups overlapping with much better alignments to a different sequence type, where "much better" was defined as a bitscore difference of at least 33. Following resolution of conflicts, VH and CH alignments underwent a second filtering step of increased stringency, requiring a minimum E-value of 10^{-10} to be retained.

Following alignment filtering, scaffolds containing surviving alignments to at least two distinct segment types (where VH, JH, and each type of constant-region exon each counted as one segment type), or alignments to one segment type covering at least 1 % of the scaffold's total length were retained as potential locus scaffolds. To reduce computational runtime spent processing irrelevant sequence on long scaffolds, each candidate scaffold so identified was trimmed to 100kb before the first putative gene segment and 100kb after the last one; in the case of *Nothobranchius furzeri* and *Xiphophorus maculatus*, these ranges were further reduced following more thorough segment characterisation (see below).

The exact set of reference sequences used for this extraction process differed depending on the genome being analysed. For *Nothobranchius furzeri*, the reference sequences extracted from medaka, stickleback and zebrafish were used; for *Xiphophorus maculatus*, gene segments inferred for *N. furzeri* were also included; and for other species, the reference sequences plus those inferred for both *N. furzeri* and *X. maculatus* were used.

In the case of both *Nothobranchius furzeri* and *Xiphophorus maculatus*, a single chromosome (chromosome 6 in *N. furzeri*, chromosome 16 in *X. maculatus*) was identified as bearing the *IGH* locus in that species. In the case of *X. maculatus*, this was the only segment-bearing scaffold identified in the genome, and the completed locus sequence was obtained by simply trimming the chromosomal sequence at either end of the segment-bearing region. In contrast, multiple scaffolds from the *N. furzeri* genome were also identified as bearing at least one potential *IGH* segment (Table S2). In order to identify which of these were in fact part of the locus and integrate them into a contiguous sequence, BAC candidates identified and assembled as described in Section A.1 were incorporated into the assembly.

To do this, all assembled BAC inserts were screened for *IGH* locus segments in the same manner described for genome scaffolds (Section A.3). Passing BACs (Table S3) were aligned to the candidate genome scaffolds with BLASTN, using the same parameters specified in Section A.1 and discarding alignments with length less than 500 bp and percentage identity less than 95%. Aligned BACs and scaffolds were then integrated manually together, giving priority in the event of a sequence conflict to (i) any sequence containing a gene segment missing from the other, and (ii) the genome scaffold sequence if neither sequence contained such a segment. Apart from cases where the genome assembly contained a gap, the former condition only occured once: a region containing two VH segments, IGH1V1-06 and IGH1V4-02p, was present in multiple BACs but not on the genome assembly (Table S7 and Fig. S7), and this region was included in the locus assembly.

BACs and scaffolds which could not be integrated into the locus sequence were discarded, as were some BACs considered redundant with other BACs; in total, four (out of six) candidate scaffolds and six (out of eleven) candidate BAC inserts were discarded this way (Tables S2 and S3).

A.4 Characterising gene segments

Detailed characterisation of *IGH* gene segments was performed on finished *IGH* locus sequences for *Xiphophorus maculatus* and *Nothobranchius furzeri*, and on isolated candidate scaffolds for other species, using the same reference segment databases used to identify candidate scaffolds for that species in Section A.3. The specific methods used depended on segment type.

A.4.1 VH

To identify VH segments on newly characterised loci, reference VH segments were used to construct a multiple-sequence alignment with PRANK[26]:

```
prank -d=<reference_vh_db> -o=<output_path> -gaprate=0.00001 -gapext=0.00001
    ↪ -F -termgap
```

The resulting alignment was used as an input to NHMMER[53], which constructs a Hidden Markov Model from a multiple-sequence alignment and uses it to identify matching sequences in a reference sequence:

```
nhmmer --dna --notextw --tblout <output_path> -T 80 <vh_alignment>
    ↪ <locus_sequence_path>
```

where `-T 80` specified the minimum alignment score required to report a match. The resulting match table was used to identify candidate ranges in the locus sequence corresponding to VH segments; these ranges were extended by 9bp at either end to account for boundary errors, and the corresponding nucleotide sequences were extracted to a FASTA file. Each sequence was then checked and refined manually: 3' ends were identified by the start of the RSS heptamer sequence (consensus CACAGTG[54]), if present, while 5' ends and FR/CDR boundaries were identified using IMGT/DomainGapAlign[55] with the default settings. Where necessary, IMGT/DomainGapAlign was also used to IMGT-gap the VH segments in accordance with the IMGT unique numbering[56].

An initial amino-acid sequence for each VH segment was produced automatically from the extracted nucleotide sequence by identifying the reading frame which minimised the number of stop codons in the sequence; this worked well for most segments. VH amino-acid sequences were then refined (and in a few cases re-translated) using the manually-refined nucleotide sequences, including end-refinement and FR/CDR boundary identification.

Following extraction and manual curation, VH segments were grouped into families based on their pairwise sequence identity. In order to assign segments to families, the nucleotide sequence of each VH segment in a locus was aligned to every other segment using Needleman-Wunsch global alignment, as implemented in the Biostrings R package (gap-opening penalty 10, gap-extention penalty 4, default substitution matrix), and the resulting matrix of pairwise sequence identities was used to perform single-linkage hierarchical clustering on the VH segments. The resulting dendrogram was cut at 80 % sequence identity to obtain VH families. These families were then numbered based on the order of the first-occurring VH segment from that family in the first *IGH* sublocus in which the family is represented, and each VH segment was named based on its parent sublocus, its family, and its order among elements of that family in that sublocus (Table S9 and Tables S16 to S20).

A.4.2 JH

As with VH segments, JH segments were identified by building a multiple-sequence alignment with PRANK and using it to construct an HMM with NHMMER; the parameters used were the same as for VH segments, except that there was no minimum score for NHMMER to report a sequence match (`-T 0` instead of `-T 80`). The resulting sequence ranges were extended by 20 bp on either end and extracted into FASTA format. These sequences were then trimmed automatically by identifying the RSS heptamer sequence at the 5' end and the splice junction motif (GTA) at the 3' end, then checked and refined manually. The JH segments were translated based on the reading frame of the conserved tryptophan (W118), and named based on their order within their parent sublocus and, where applicable, on whether they were upstream of *IGHZ* or *IGHM* constant regions (Tables S13 and S24).

A.4.3 DH

Unlike VH and JH gene segments, DH segments are too short and unstructured to be found effectively using an HMM-based search strategy. Instead, DH segments in assembled loci were located using their distinctive pattern of flanking recombination signal sequences: an antisense RSS in 5', then a short D-segment, then a sense RSS in 3'. Potential matches to this pattern were searched for using FUZZNUC from the EMBOSS collection of bioinformatics tools[58], with a high error tolerance to account for deviations from the conserved sequence in either or both of the RSSs:

```
fuzznuc -pattern 'GGTTTTTGTN(10,14)CACTGTGN(1,25)CACAGTGN(10,14)ACAAAAACC'
    ↪ -pmismatch 8 -rformat gff -outfile <output_path> <locus_sequence_path>
```

This generated a GFF file of permissive matches, representing potential DH segments; these were then arranged by sequence co-ordinate, and higher-mismatch candidates overlapping with a lower-mismatch alternative were discarded.

Automatic orientation of DH segments based on their own sequence is challenging, as the segments themselves have no clear conserved structure and the flanking RSSs are rotationally symmetric. To overcome this problem and orientate the DH segments on the locus, the table of DH candidate ranges was combined with previously-identified VH and JH ranges. Each DH candidate was then orientated based on the orientations of its flanking segments: segments with an oriented segment immediately upstream or downstream adopted the orientation of that segment, while segments with contradictory orientation information were discarded. This process was repeated until all DH candidates had either been orientated or discarded.

After orientation, the DH ranges were used to extract DH sequences in FASTA format from the locus sequence; these sequences then underwent a second, more stringent filtering step, in which sequences lacking the most conserved positions in each RSS[54] were discarded:

```
grep -B 1 '[ACTG]\{{25,27\}}TG[ACTG]\{{1,25\}}CA[ACTG]\{{25,27\}}' <dh_fasta>
    ↪ | sed '/^--$'/d > <output_fasta>
```

Finally, the identified DH candidates were checked manually, candidates without good RSS sequences were discarded, and flanking RSS sequences were trimmed to obtain the DH segment sequences themselves. As with the JH segments, these were numbered based on their order within their parent sublocus and, when applicable, on whether they were upstream of *IGHZ* or *IGHM* constant regions (Tables S10 and S22).

A.4.4 CH

To detect and identify constant-region exons in the characterised loci, constant-region nucleotide and protein sequences from reference species were mapped to the locus sequence using BLAST[39], in the same manner described for putative locus scaffolds in Section A.3. Following alignment of reference sequences, overlapping alignments to reference segments of the same isotype and exon number were collapsed together, keeping track of the number of collapsed alignments and the best E-values and bitscores obtained for each alignment group. Alignment groups with a very poor maximum E-value (> 0.001) were discarded, as were groups overlapping with a much better alignment to a different isotype or exon type, where "much better" was here defined as a bitscore difference of at least 16.5. Where conflicting alignments to different isotypes or exon types co-occurred without a sufficiently large difference in bitscore, both alignment groups were retained for manual resolution of exon identity.

Following resolution of conflicts, alignment groups underwent a second filtering step of increased stringency, requiring a minimum E-value of 10^{-8} and at least two aligned reference exons over all reference species to be retained. Each surviving alignment group was then converted to a sequence range, extended by 10 bp at each end to account for truncated alignments failing to cover the ends of the exon, and used to extract the corresponding exon sequence into FASTA format. These sequences then underwent manual curation to resolve conflicting exon identities, assign exon names and perform initial end refinement based on putative splice junctions.

In order to validate intron/exon boundaries and investigate splicing behaviour among *IGH* constant-region exons in *N. furzeri* and *X. maculatus*, published Illumina RNA-sequencing reads (Table S5) were aligned to the annotated locus using STAR[25]. In both cases, reads files from multiple individuals were concatenated and aligned together, in order to make the intron/exon boundary changes in mapping behaviour as clear as possible.

Before aligning the RNA-seq reads, each locus underwent basic repeat masking, using the built-in zebrafish repeat parameters from `RepeatMasker`[63]:

```
RepeatMasker -species danio -dir <masked_locus_dir> -s <unmasked_locus_path>
```

After masking, a STAR genome index was generated from each locus:

```
STAR --runMode genomeGenerate --genomeDir <star_index_directory_path>
    ↪ --genomeFastaFiles <masked_locus_path> --genomeSAindexNbases <sa_index>
```

where the `--genomeSAindexNbases` option determined the size of the suffix-array index and was dependent on the length of the reference sequence being indexed:

$$\text{SA index size (bits)} = \left\lfloor \frac{\log_2(\text{length of reference sequence})}{2} - 1 \right\rfloor \qquad (1)$$

Following index generation, the RNA-seq reads were mapped to the generated index as follows:

```
STAR --genomeDir <star_index_directory_path> --readFilesIn <input_reads>
    ↪ --outFilterMultimapNmax 5 --alignIntronMax 10000 --alignMatesGapMax
    ↪ 10000 --outFilterMatchNminOverLread 0.98
```

where the `--outFilterMultimapNmax` option excludes read pairs mapping to more than five distinct co-ordinates in the reference sequence, the `--alignIntronMax` option excludes reads spanning predicted introns of more than 10 kb, the `--alignMatesGapMax` option excludes read pairs mapping more than 10 kb apart, and the -lstinlineoutFilterMatchNminOverLread option excludes read pairs with more than 2% deviation from the reference sequence. Following alignment, the resulting SAM files were processed into sorted, indexed BAM files using `SAMtools`[48] and visualised with Integrated Genomics Viewer (`IGV`[49]) to determine intron/exon boundaries of predicted exons, as well as the major splice isoforms present in each dataset.

In order to reduce time and memory requirements for generating alignment figures (Fig. 3 and Fig. S4 to S6), secondary alignments were performed on truncated loci consisting only of the *IGHM/D* or (where present) *IGHZ* constant regions, plus a few flanking kilobases on each side. In these cases, the additional parameters constraining multimapping, intron length and mate distance were not necessary due to the much shorter and less-repetitive reference sequence. Coverage and Sashimi plots were generated using Gviz; with the exception of reads splicing $C_\mu 1$ to the start of the *IGHD* constant region, only splice junctions covered by some threshold number of reads were shown, with the threshold determined by the size of the read dataset and the expression level of the isotype in question; see Table S8 for more information.

For species other than *N. furzeri* or *X. maculatus*, intron/exon boundaries were predicted manually based on BLASTN and BLASTP alignments to closely-related species (using the same parameters specified in Section A.3) and the presence of conserved splice-site motifs (AG at the 5' end of the intron, GT at the 3' end[51]). In cases where no 3' splice site was expected to be present (e.g. for CM4 or TM2 exons), the nucleotide exon sequence was terminated at the first canonical polyadenylation site (AATAAA if present, otherwise one of ATTAAA, AGTAAA or TATAAA[52]), while the amino-acid sequence was terminated at the first stop codon. In many cases, it was not possible to locate a TM2 exon due to its very short conserved coding sequence (typically only 2 to 4 amino-acid residues[9,11]).

A.5 Cross-sublocus sequence comparison

Synteny between subloci in the *N. furzeri* locus (Fig. S2a) was analysed using the standard synteny pipeline from the `R` package `DECIPHER`[61], which searches for chains of exact *k*-mer matches within two sequences:

```
DBPath <- tempfile()
DBConn <- dbConnect(SQLite(), DBPath)

Seqs2DB(seqs = <sublocus_1_sequence>, type = "XStringSet", dbFile = DBConn,
    ↪ identifier = "IGH1", verbose = FALSE)
Seqs2DB(seqs = <sublocus_2_sequence>, type = "XStringSet", dbFile = DBConn,
    ↪ identifier = "IGH2", verbose = FALSE)

dbDisconnect(DBConn)

SyntenyObject <- FindSynteny(dbFile = DBPath, verbose = FALSE)
```

Cross-locus sequence comparisons between gene segments were performed analogously to the comparisons involved in VH family assignment, with `pairwiseAlignment` and `pid` from Biostrings.

A.6 Phylogenetic trees

The cladogram topology in Fig. 1 and 5a was obtained from the comprehensive teleost phylogeny of Hughes *et al.*[59], while additional, higher-resolution information on the interrelationships of African killifishes missing from that tree was provided by Cui *et al.*[31]. Annotations (e.g. of clade membership or isotype status) were added using `tidytree`[64].

To obtain the molecular phylogram of *IGHZ* constant regions, the $C_\zeta 1$-4 exons from each *IGHZ* constant region were concatenated into a single sequence. In the event of partial constant regions missing one or more C_ζ exons, the remaining exons were concatenated together in the usual order. Concatenated sequences from all species were then collated into a single FASTA file, and entries with completely identical sequences were collapsed together into a single FASTA sequence. A multiple-sequence alignment of the remaining sequences was then constructed with PRANK:

```
prank -d=<sequence_fasta> -o=<output_prefix> -DNA -termgap
```

The resulting alignment was passed to the maximum-likelihood phylogenetic inference program RAxML[27], using the SSE3-enabled parallelised version of the software, the standard GTR-Gamma nucleotide substitution model, and built-in rapid bootstrapping:

```
raxmlHPC-PTHREADS-SSE3 -f a -m GTRGAMMA -s <ch_prank_alignment> -w
    ↪ <output_dir> -N 1000 -x 53067 -p 106939 -n <output_suffix>
```

Finally, the bootstrap-annotated `RAxML_bipartitions` file was inspected and rooted manually in Figtree[65], before being annotated and visualised in R with tidytree and ggtree, respectively. During tree visualisation, nodes with bootstrap support of less than 65 % were collapsed into polytomies.

A.7 Data and code availability

All genomes used in this analysis are available at the accessions specified in Table S4. Locus and gene-segment sequences for reference species (medaka, zebrafish and stickleback) are available in the supplementary information from their respective publications[9–12] and reproduced in the Supplementary Data for this article. Accessions for the RNA-sequencing reads used in the alternative splicing analysis are specified in Table S5. Raw killifish BAC sequencing reads, annotated locus sequences, variable gene segments, and constant-region exon sequences are all provided in the Supplementary Data for this article. All code used to perform the analyses in this article is available upon request.

B Supplementary figures

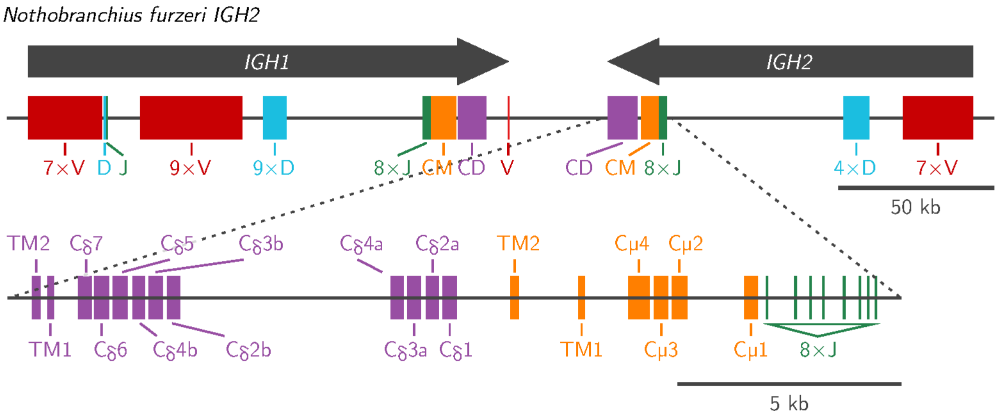

Figure S1: ***IGH2* sublocus structure in *Nothobranchius furzeri***. Arrangement of VH, DH, JH and constant regions on the *N. furzeri IGH* locus, indicating the two subloci *IGH1* and *IGH2* and the detailed exon composition of the *IGH2* constant regions.

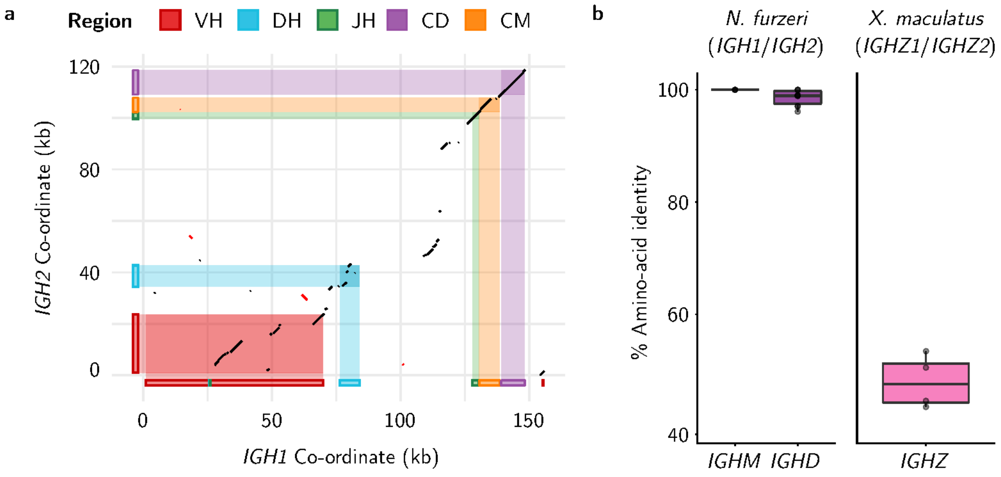

Figure S2: **Internal similarity of *IGH* loci a**, Synteny dot plot of sequential best matches between *N. furzeri IGH1* and *IGH2* sequences, with gene-segment regions in each sublocus indicated by coloured rectangles along each axis. **b**, Boxplots of percentage amino-acid sequence identity between corresponding C_μ and C_δ exons in *N. furzeri IGH1* vs *IGH2* subloci (left) or between corresponding C_ζ exons in *X. maculatus IGHZ1* vs *IGHZ2* constant regions (right).

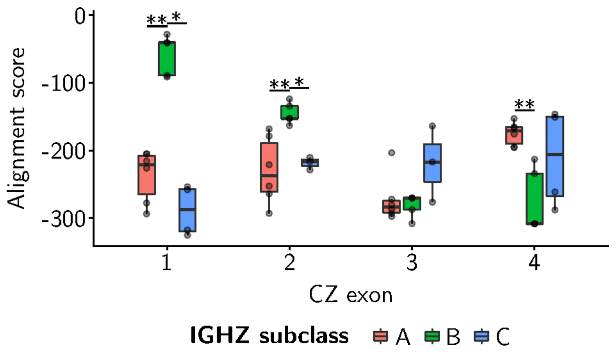

Figure S3: ***Pachypanchax playfairii IGHZ* is composed of exons from multiple ancestral subclasses.** Boxplots of Needleman-Wunsch alignment scores between the amino-acid sequences of *Pachypanchax playfairii* C_ζ exons and those of equivalent exons from seven other *IGHZ*-bearing cyprinodontiform species, demonstrating the differing affinity of different *P. playfairii* exons for each of the three *IGHZ* subclasses. Less negative scores indicate a stronger alignment. Pairwise p-values were computed using nonparametric Mann-Whitney U tests ($* : 0.01 < p \leq 0.05$; $** : 0.001 < p \leq 0.01$).

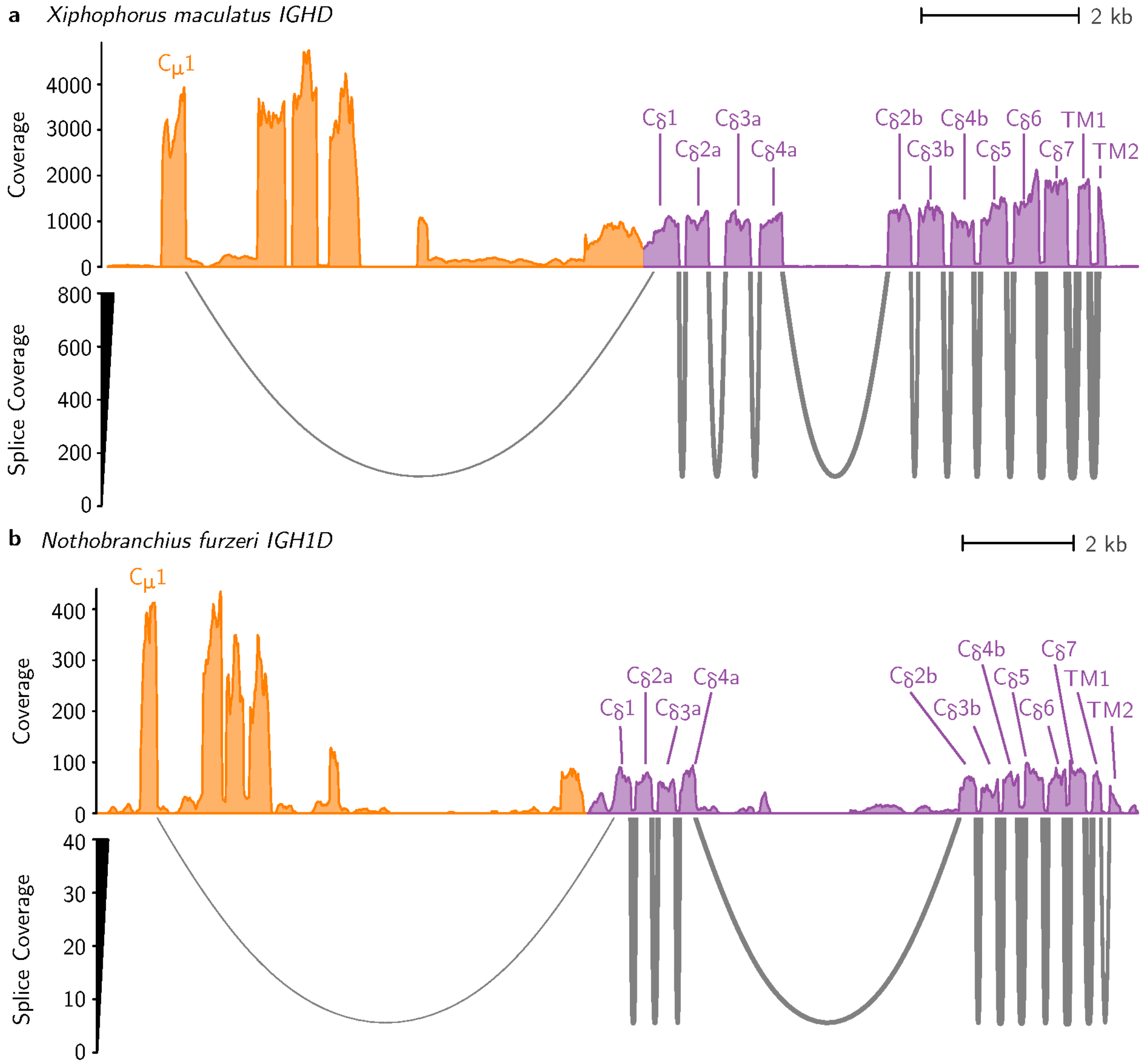

Figure S4: Read coverage and Sashimi plots showing alignment and splicing behaviour of RNA sequencing reads aligned to the *IGHD* constant regions of **a**, *Xiphophorus maculatus* and **b**, *Nothobranchius furzeri*, showing the chimeric splicing of $C_{\mu}1$ to the start of the *IGHD* constant region in both species.

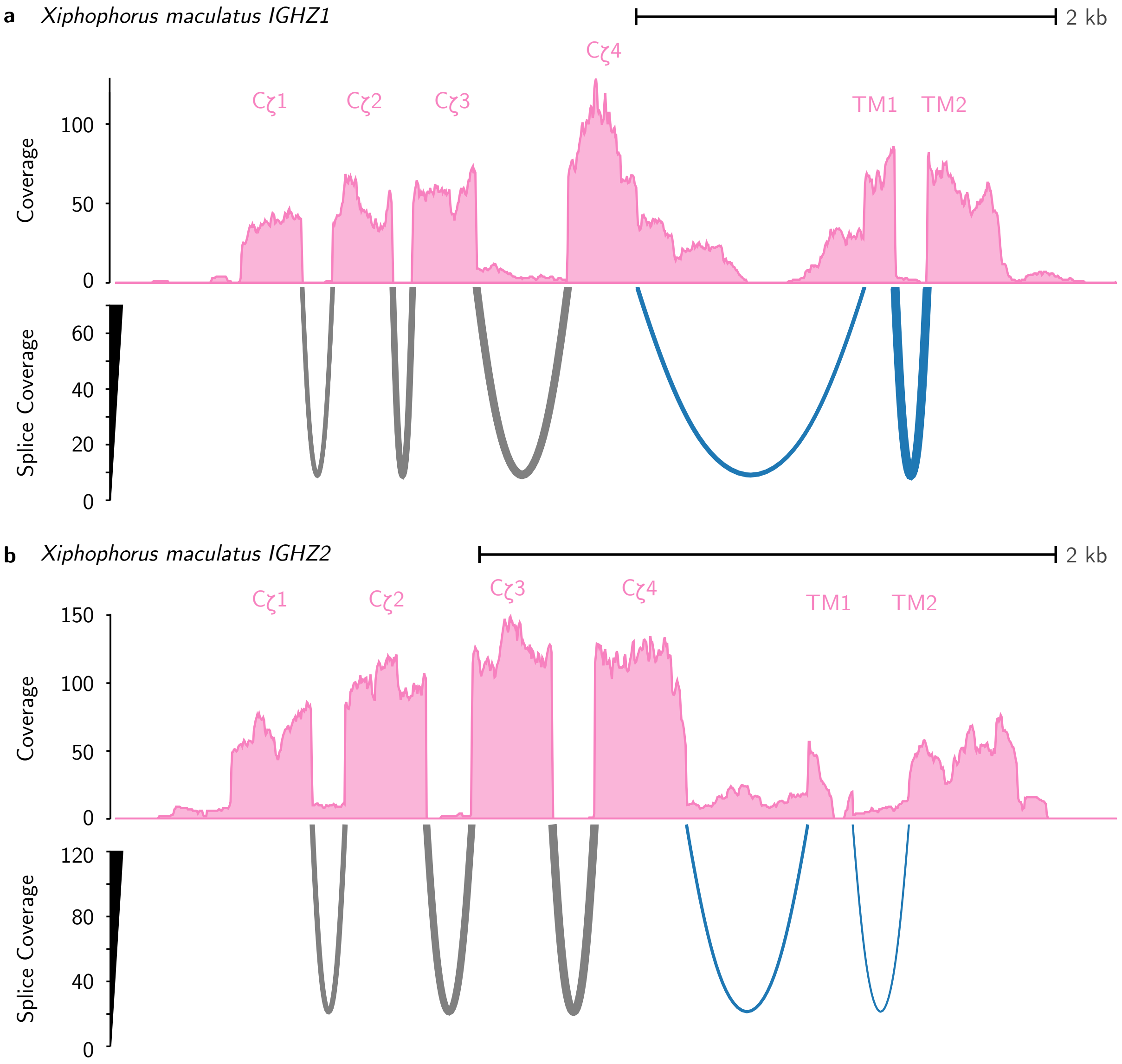

Figure S5: Read coverage and Sashimi plots showing alignment and splicing behaviour of RNA sequencing reads aligned to the (a) *IGHZ1* and (b) *IGHZ2* constant regions of *Xiphophorus maculatus*, showing the alternative splicing of secreted (grey) and transmembrane (grey+blue) isoforms in both cases. Note the apparent expression of a post-splice-site secretory tail after Cζ4 in *IGHZ1* but not *IGHZ2*.

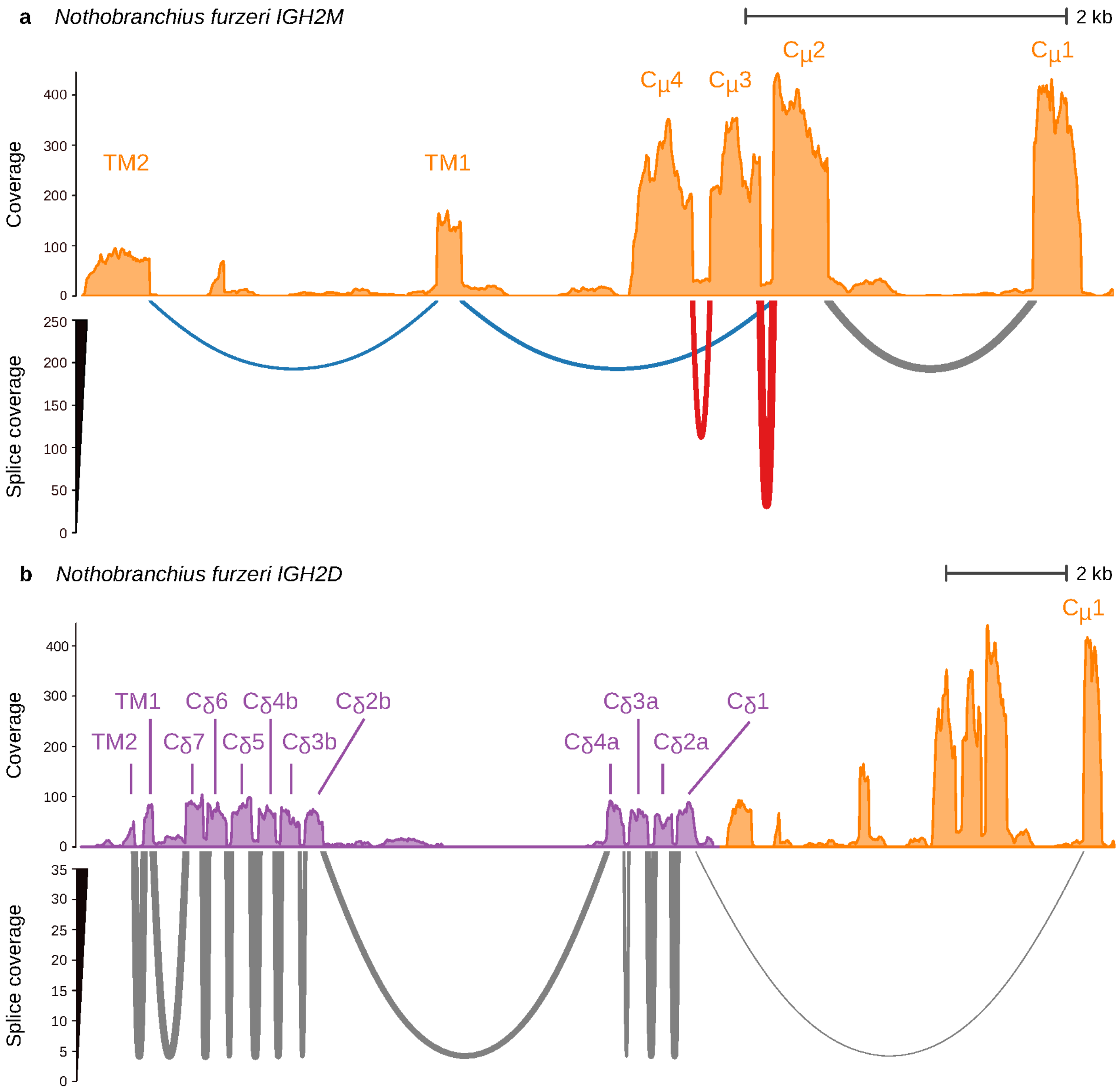

Figure S6: Read coverage and Sashimi plots showing alignment and splicing behaviour of RNA sequencing reads aligned to the constant regions of *N. furzeri IGH2*: (a) *IGH2M* and (b) *IGH2D*.

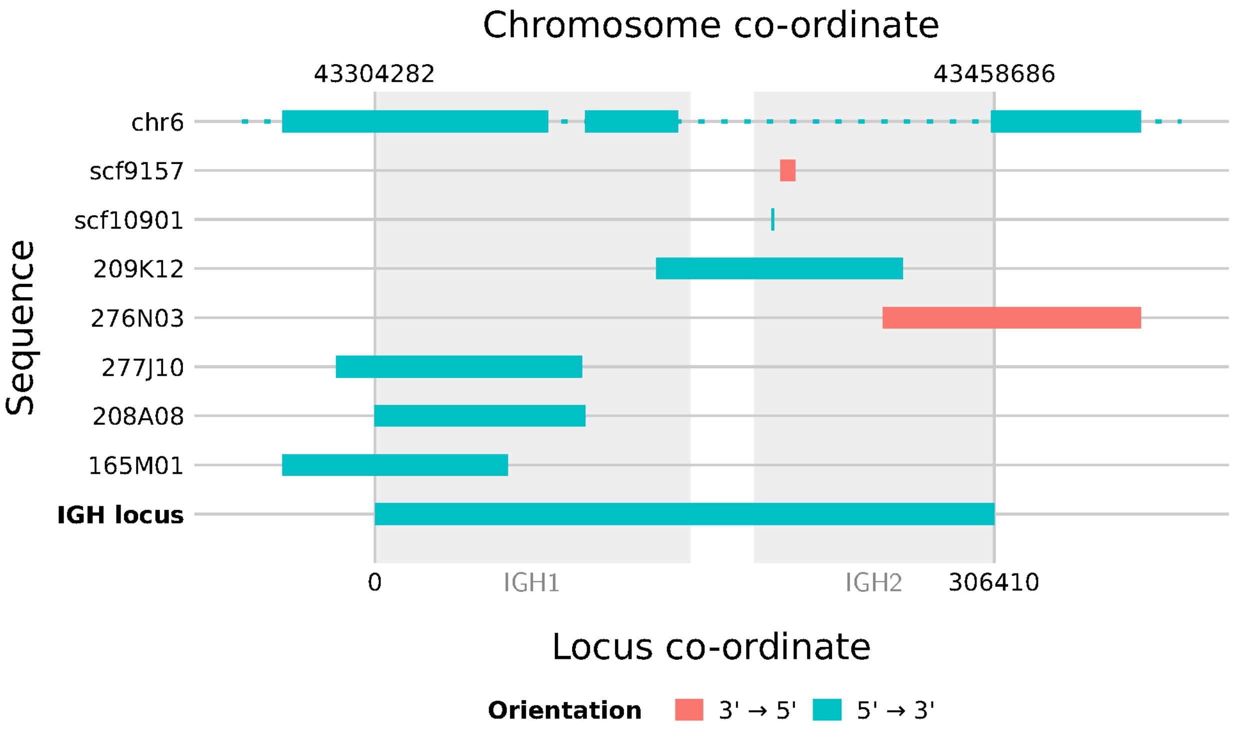

Figure S7: Assembling the *Nothobranchius furzeri IGH* locus: Schematic of genome scaffolds and BAC inserts contributing to the *Nothobranchius furzeri IGH* locus sequence, with their corresponding place within the locus sequence (bottom axis). Internal gaps with dotted lines indicate regions on chromosome 16 with no corresponding locus sequence, as a result of intercalation of BAC or scaffold sequences. Grey shaded regions indicate the positions of the two subloci *IGH1* and *IGH2*; note the insert sequence of BAC 209K12 spanning both subloci.

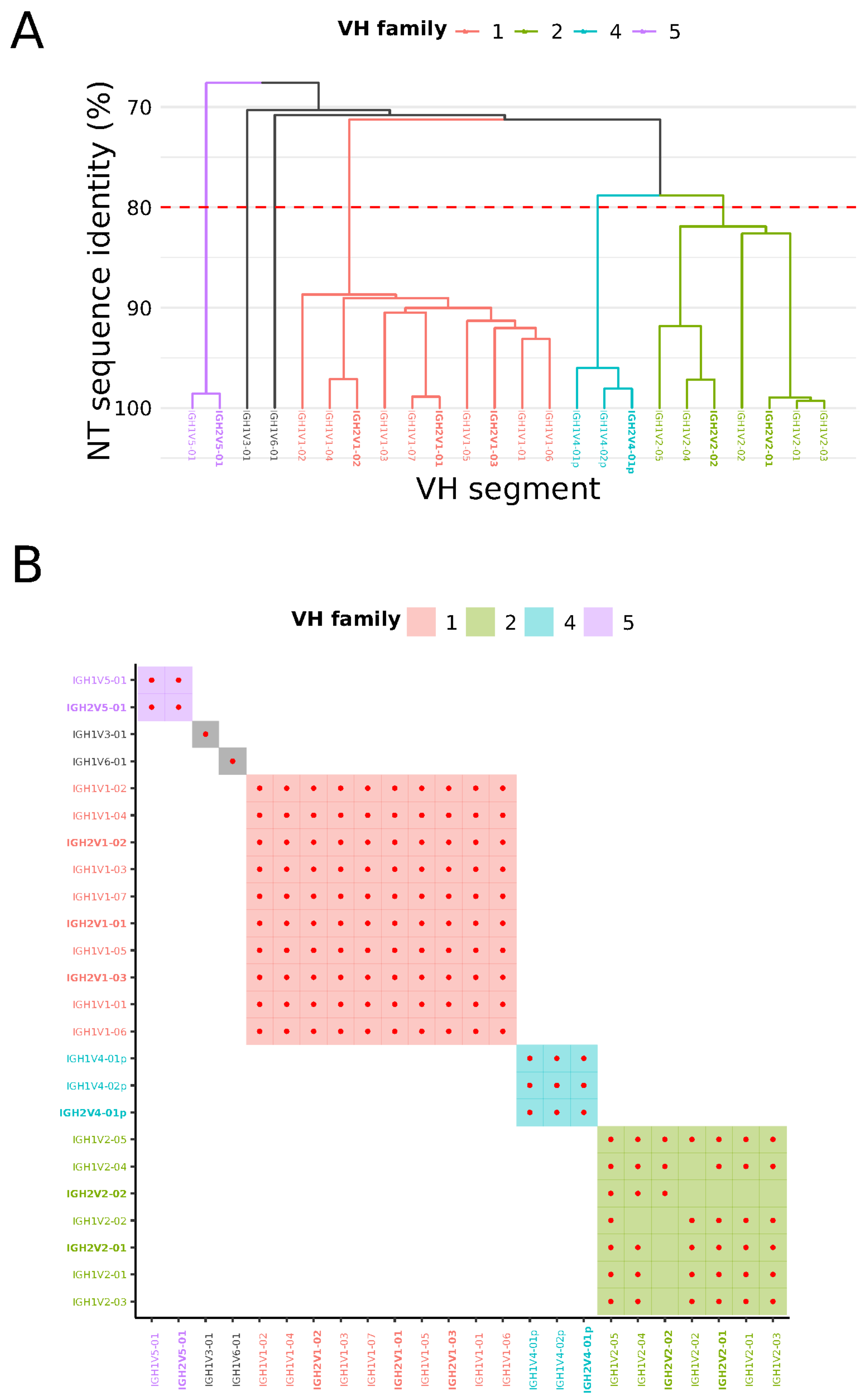

Figure S8: VH families in the *Nothobranchius furzeri IGH* locus: (A) Dendrogram of sequence similarity of VH segments in the *Nothobranchius furzeri IGH* locus, arranged by single-linkage clustering on nucleotide sequence identity. The red line indicates the 80 % cutoff point for family assignment. (B) Heatmap of family relationships among *Nothobranchius furzeri* VH segments, with shaded squares indicating families and red dots indicating pairwise nucleotide sequence identity of at least 80 %. In both subfigures, VH families containing multiple segments are uniquely coloured, single-segment families are in grey, and segments from the *IGH2* sublocus are displayed in boldface.

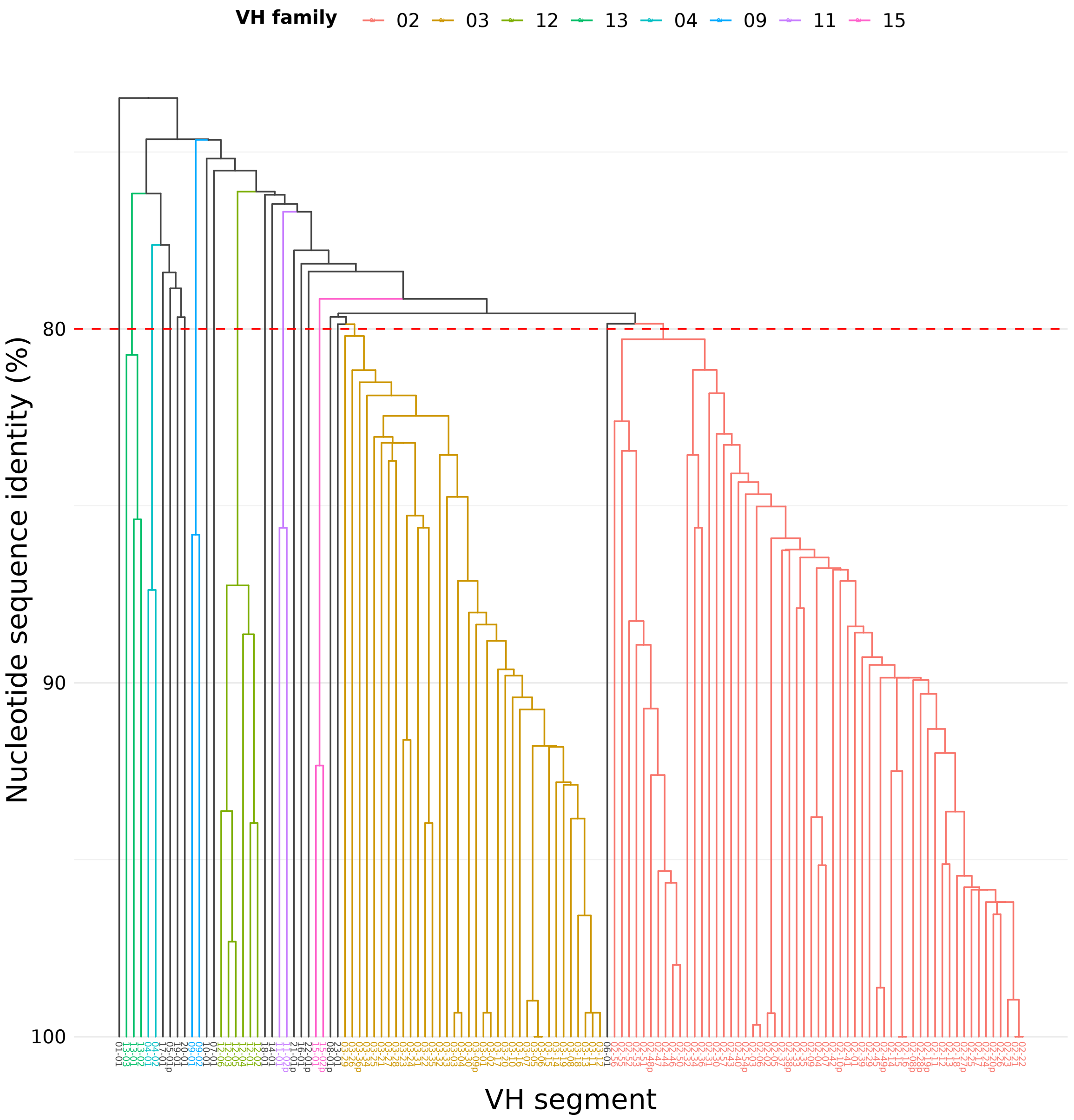

Figure S9: Dendrogram of VH families in the *Xiphophorus maculatus IGH* locus: Dendrogram of sequence similarity of VH segments in the *Xiphophorus maculatus* locus, arranged by single-linkage clustering on nucleotide sequence identity. The red line indicates the 80 % cutoff point for family assignment, while branch colour indicates family membership: VH families containing multiple segments are uniquely coloured, while single-segment families are in grey.

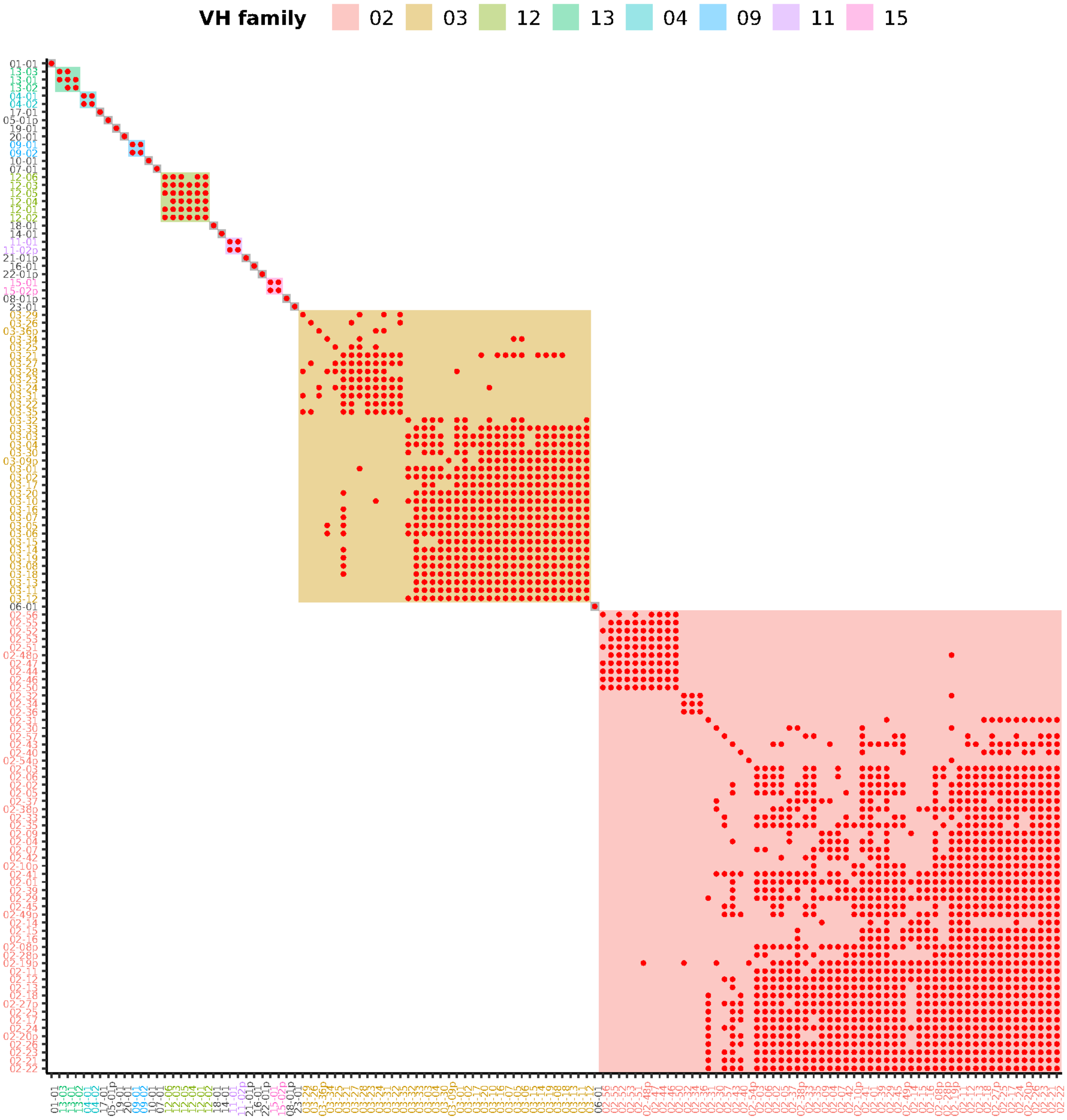

Figure S10: Heatmap of VH families in the *Xiphophorus maculatus IGH* locus: Heatmap of family relationships among *Xiphophorus maculatus* VH segments, with coloured shading indicating families and red dots indicating pairwise nucleotide sequence identity of at least 80%. VH families containing multiple segments are uniquely coloured, while single-segment families are in grey.

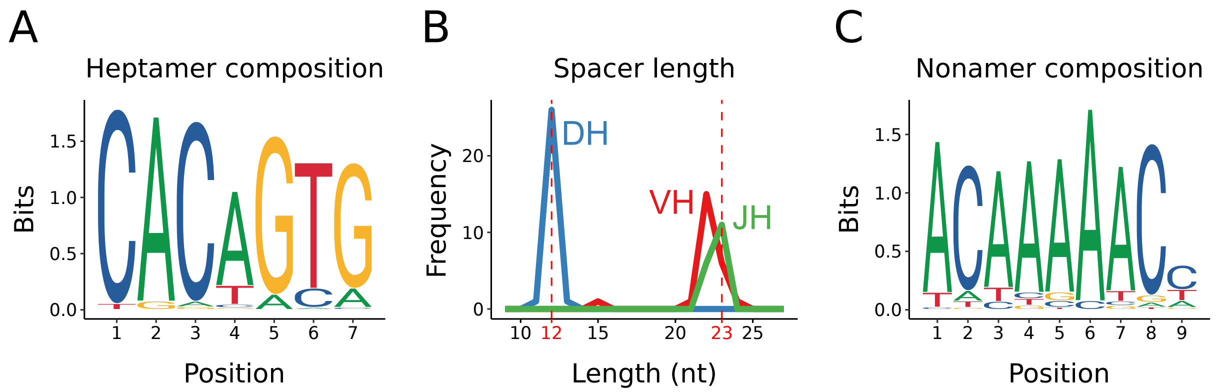

Figure S11: Recombination signal sequences in *Nothobranchius furzeri IGH*: (A) Sequence composition of conserved heptamer sequences across all *Nothobranchius furzeri* heavy-chain RSSs; (B) length distribution of unconserved spacer sequences in *Nothobranchius furzeri* heavy-chain RSSs; (C) sequence composition of conserved heptamer sequences across all *Nothobranchius furzeri* heavy-chain RSSs.

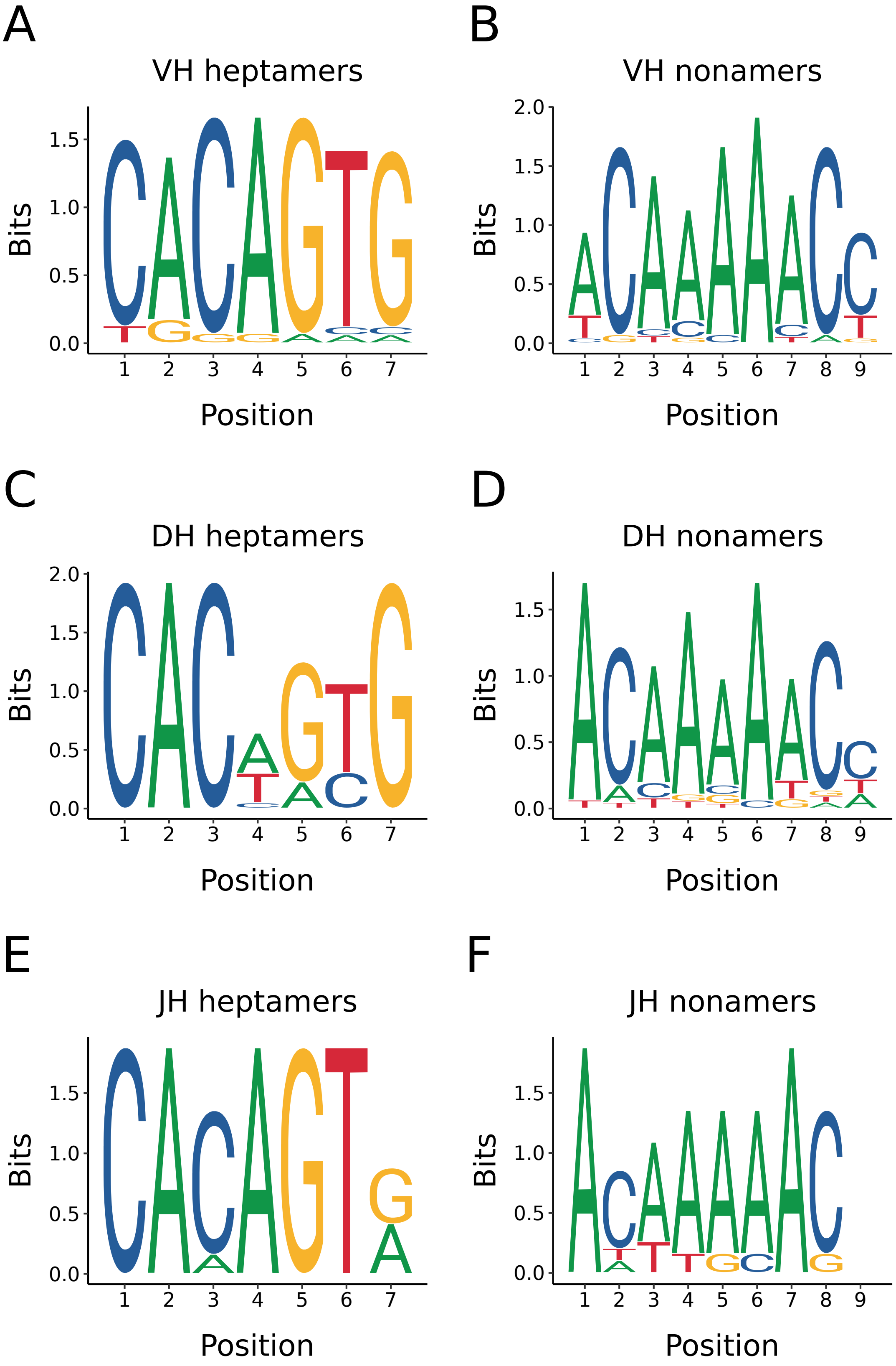

Figure S12: *Nothobranchius furzeri* recombination signal sequences by segment type: Sequence composition of conserved heptamer (A,C,E) and nonamer (B,D,F) sequences from *Nothobranchius furzeri* heavy-chain RSSs associated with VH (A,B), DH (C,D) or JH (E,F) gene segments.

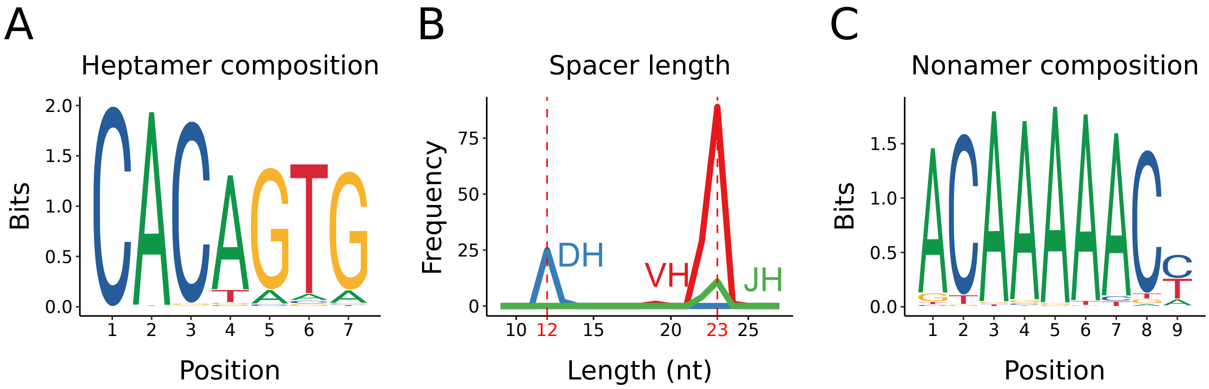

Figure S13: Recombination signal sequences in the *Xiphophorus maculatus IGH* locus: (A) Sequence composition of conserved heptamer sequences across all *Xiphophorus maculatus* heavy-chain RSSs; (B) length distribution of unconserved spacer sequences in *Xiphophorus maculatus* heavy-chain RSSs; (C) sequence composition of conserved heptamer sequences across all *Xiphophorus maculatus* heavy-chain RSSs.

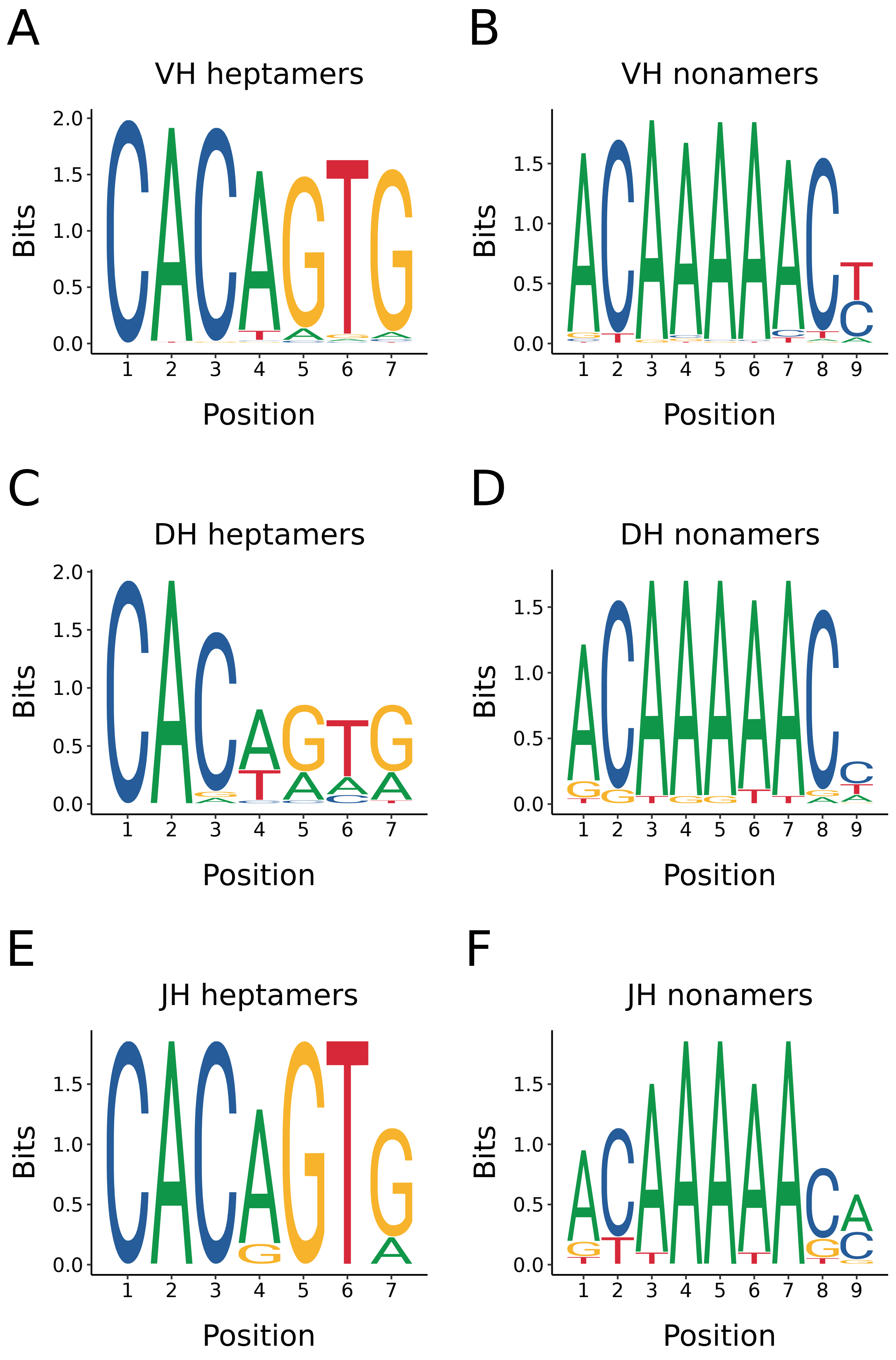

Figure S14: *Xiphophorus maculatus* recombination signal sequences by segment type: Sequence composition of conserved heptamer (A,C,E) and nonamer (B,D,F) sequences from *X. maculatus* heavy-chain RSSs associated with VH (A,B), DH (C,D) or JH (E,F) gene segments.

C Supplementary tables

Table S1: Versions of software and R packages used in computational analyses

Program	Version
ape	5.2
Basemount	0.15.96.2154
Biostrings	2.50.1
BLAST	2.7.1
Bowtie 2	2.2.6
BSgenome	1.50.0
DECIPHER	2.10.0
EMBOSS (FUZZNUC)	6.6.0
FigTree	1.4.2
HMMER	3.2
GenomicRanges	1.34.0
ggtree	1.14.4
ggseqlogo	0.1
Gviz	1.27.6
IGV	2.3.68
IMGT/DomainGapAlign	4.9.2
PRANK	v.170427
Primer3	2.3.6
QuorUM	1.0.0
R	3.5.2
RAxML	8.2.12
RepeatMasker	4.0.6
SAMtools	1.9
sed	4.2.2
seqtk	1.3
Snakemake	5.3.0
SPAdes	3.6.1
SSPACE	3.0
STAR	2.5.2b
tidytree	0.2.0
tidyverse	1.2.1
Trimmomatic	0.32

Table S2: *N. furzeri* genome scaffolds containing putative *IGH* locus fragments

Scaffold	Total length (kb)	V	J	C_μ	C_δ	C_ζ	Included in locus?
chr6	6195.6	15	7	5	11	0	Yes
scf10901	1.4	0	0	0	3	0	Yes
scf21863	13.5	1	0	0	0	0	No
scf35954	16.3	3	0	0	0	0	No
scf36277	18.9	2	1	0	0	0	No
scf37083	17.7	1	0	0	0	0	No
scf9157	7.2	0	7	4	0	0	Yes

Table S3: *N. furzeri* BAC-library inserts containing putative *IGH* locus fragments

BAC ID	Total length (kb)	Approximate coverage (×)[a]	V	J	C_μ	C_δ	C_ζ	Included in locus?
154G24	106.6	1514	17	1	0	0	0	No
162F04	119.4	1612	5	1	0	0	0	No
165M01	110.7	1974	15	1	0	0	0	Yes
206K13	106.7	2935	17	1	0	0	0	No
208A08	103.2	1035	17	1	0	0	0	Yes
209K12	133.0	1029	1	8	4	20	0	Yes
220O06	104.8	982	4	1	0	0	0	No
223M21	99.3	1593	17	1	0	0	0	No
248A22	47.3	3837	7	0	0	0	0	No
276N03	127.9	1301	7	0	0	0	0	Yes
277J10	120.8	1295	17	1	0	0	0	Yes

[a] Total length of all sequencing reads, following read trimming and initial filtering to exclude *E. coli* genomic contamination, divided by insert length.

Genus	Species	Common Name	Bioproject/GenBank Accession
Nothobranchius	*furzeri*	Turquoise killifish	JAAVVJ010000000
Xiphophorus	*maculatus*	Southern platyfish	GCA_002775205.2
Austrofundulus	*limnaeus*	–	GCA_001266775.1
Fundulus	*heteroclitus*	Mummichog	GCA_000826765.1
Poecilia	*formosa*	Amazon molly	GCA_000485575.1
Poecilia	*reticulata*	Guppy	GCA_000633615.1
Cyprinodon	*variegatus*	Sheepshead minnow	GCA_000732505.1
Kryptolebias	*marmoratus*	Mangrove rivulus	GCA_001649575.1
Aphyosemion	*australe*	Lyretail panchax	GCA_006937985.1
Callopanchax	*toddi*	–	GCA_006937965.1
Pachypanchax	*playfairii*	Golden panchax	GCA_006937955.1
Nothobranchius	*orthonotus*	Spotted killifish	GCA_006942095.1
Oryzias	*latipes*	Medaka	GCA_002234675.1

[a] Willemsen *et al.*[38]

Table S4: Genome assemblies used to identify *IGH* locus sequences in cyprinodontiform fishes

Table S5: RNA-sequencing datasets used for *IGH* locus characterisation

**Species**	*N. furzeri*	*X. maculatus*
**Tissues**	Gut	Various[a]
**BioProject Accession**	PRJNA379208	PRJNA420092
**SRA Run Accessions**	SRR5344350<br>SRR5344343<br>SRR5344344<br>SRR5344345<br>SRR5344346<br>SRR5344347<br>SRR5344348<br>SRR5344349<br>SRR5344350	SRR6327069<br>SRR6327070<br>SRR6327071<br>SRR6327072<br>SRR6327073<br>SRR6327074<br>SRR6327075<br>SRR6327076<br>SRR6327077<br>SRR6327078<br>SRR6327079<br>SRR6327080<br>SRR6327081<br>SRR6327082<br>SRR6327083<br>SRR6327084<br>SRR6327085<br>SRR6327086<br>SRR6327087<br>SRR6327088<br>SRR6327089<br>SRR6327090<br>SRR6327091<br>SRR6327092<br>SRR6327093<br>SRR6327094
**Source**	[66]	Citation not given in PioProject

[a] Tissues used for *X. maculatus* RNA-sequencing included brain, heart, liver, gut, skin or whole fish; see BioProject entry for details.

Table S6: Co-ordinate table of constant-region exons in the *N. furzeri IGH* locus

Name	Isotype	Start	End	Length	Strand
IGH1M-1	M	130848	131144	297	+
IGH1M-2	M	131971	132312	342	+
IGH1M-3	M	132394	132705	312	+
IGH1M-4	M	132816	133288	473	+
IGH1M-TM1	M	134262	134413	152	+
IGH1M-TM2	M	138431	138819	389	+
IGH1D-1	D	139381	139689	309	+
IGH1D-2A	D	139774	140064	291	+
IGH1D-3A	D	140178	140489	312	+
IGH1D-4A	D	140572	140853	282	+
IGH1D-2B	D	145613	145909	297	+
IGH1D-3B	D	146000	146311	312	+
IGH1D-4B	D	146398	146676	279	+
IGH1D-5	D	146795	147124	330	+
IGH1D-6	D	147210	147527	318	+
IGH1D-7	D	147598	147885	288	+
IGH1D-TM1	D	148016	148164	149	+
IGH1D-TM2	D	148323	148504	182	+
IGH2D-TM2	D	187624	187803	180	-
IGH2D-TM1	D	187963	188111	149	-
IGH2D-7	D	188658	188945	288	-
IGH2D-6	D	189016	189333	318	-
IGH2D-5	D	189419	189748	330	-
IGH2D-4B	D	189867	190145	279	-
IGH2D-3B	D	190232	190543	312	-
IGH2D-2B	D	190636	190932	297	-
IGH2D-4A	D	195644	195925	282	-
IGH2D-3A	D	196008	196319	312	-
IGH2D-2A	D	196433	196723	291	-
IGH2D-1	D	196808	197116	309	-
IGH2M-TM2	M	198315	198506	192	-
IGH2M-TM1	M	199834	199985	152	-
IGH2M-4	M	200953	201425	473	-
IGH2M-3	M	201536	201847	312	-
IGH2M-2	M	201929	202270	342	-
IGH2M-1	M	203549	203845	297	-

Table S7: Placement of VH segments on sequences contributing to the *N. furzeri IGH* locus (Fig. S7 and Tables S2 and S3)

VH	chr6	277J10	208A08	165M01	209K12	276N03
IGH1V1-01	✓	✓	✓	✓	—	—
IGH1V1-02	✓	✓	✓	✓	—	—
IGH1V2-01	✓	✓	✓	✓	—	—
IGH1V1-03	✓	✓	✓	✓	—	—
IGH1V3-01	✓	✓	✓	✓	—	—
IGH1V2-02	✓	✓	✓	✓	—	—
IGH1V4-01p	✓	✓	✓	✓	—	—
IGH1V1-04	✓	✓	✓	✓	—	—
IGH1V2-03	✓	✓	✓	✓	—	—
IGH1V1-05	✓	✓	✓	✓	—	—
IGH1V5-01	✓	✓	✓	✓	—	—
IGH1V2-04	✓	✓	✓	✓	—	—
IGH1V6-01	✓	✓	✓	✓	—	—
IGH1V1-06	—	✓	✓	✓	—	—
IGH1V4-02p	—	✓	✓	—	—	—
IGH1V2-05	✓	✓	✓	—	—	—
IGH1V1-07	—	—	—	—	✓	—
IGH2V2-02	—	—	—	—	—	✓
IGH2V4-01p	—	—	—	—	—	✓
IGH2V5-01	—	—	—	—	—	✓
IGH2V1-03	—	—	—	—	—	✓
IGH2V1-02	—	—	—	—	—	✓
IGH2V2-01	—	—	—	—	—	✓
IGH2V1-01	✓	—	—	—	—	✓

Table S8: Splice coverage thresholds for Gviz Sashimi plots

Species	Constant region	Coverage threshold (reads)	Figure
N. furzeri	*IGH1M*	60	3
X. maculatus	*IGHM*	360	3
N. furzeri	*IGH1D*	30[a]	S4
X. maculatus	*IGHD*	300[a]	S4
X. maculatus	*IGHZ1*	10	S5
X. maculatus	*IGHZ2*	20	S5
N. furzeri	*IGH2M*	60	S6
N. furzeri	*IGH2D*	30[a]	S6

[a] Excluding the $C_{\mu}1$–$C_{\delta}1$ splice junction.

Name	Start	End	Length	Strand	RSS Start	Heptamer	Spacer Length	Nonamer	RSS End	RSS Length	Comment
IGH1V1-01	1252	1540	289	+	1541	CACAGTG	22	ACAAAAACC	1578	38	
IGH1V1-02	3365	3656	292	+	3657	CACAGTG	22	ACAAAAACC	3694	38	
IGH1V2-01	5907	6201	295	+	6202	CACAGAA	15	ACAAAAACT	6232	31	
IGH1V1-03	13690	13964	275	+	13965	CACAGTG	22	ACAAAAACC	14002	38	
IGH1V3-01	14862	15162	301	+	15163	CACAGTG	23	ACAAAAACC	15201	39	
IGH1V2-02	17433	17730	298	+	17731	CACAATG	23	ACAAAAACC	17769	39	
IGH1V4-01p	24566	24837	272	+	24838	CGCAGTG	22	CCACAAACC	24875	38	Nonsense mutation
IGH1V1-04	37305	37596	292	+	37597	CACAGTG	22	ACAAAAACC	37634	38	
IGH1V2-03	48845	49139	295	+	49140	CACAGTG	23	TCAAAAACT	49178	39	
IGH1V1-05	49909	50197	289	+	50198	CACAGTG	22	ACAAAAACC	50235	38	
IGH1V5-01	51710	51998	289	+	51999	CACAGTG	22	ACAAAAACT	52036	38	
IGH1V2-04	56322	56616	295	+	56617	CACAGTG	23	ACAAAAACC	56655	39	
IGH1V6-01	57465	57762	298	+	57763	CACAGTG	21	ACTAAATCT	57799	37	
IGH1V1-06	59678	59966	289	+	59967	CACAGTG	22	ACAAAAACC	60004	38	
IGH1V4-02p	68017	68288	272	+	68289	TGCAGTG	22	TCACAAACC	68326	38	Nonsense mutation
IGH1V2-05	69787	70084	298	+	70085	CACAGTG	23	ACAAAAACC	70123	39	
IGH1V1-07	155485	155763	279	+	155764	CACAGTG	22	TCAAAACCC	155801	38	
IGH2V2-02	282620	282914	295	-	282915	CACAGTG	23	ACAAAAACC	282953	39	
IGH2V4-01p	284404	284675	272	-	284676	TGCAGTG	22	TCACAAACC	284713	38	Nonsense mutation
IGH2V5-01	288808	289096	289	-	289097	CACAGTG	22	ACAGAAACT	289134	38	
IGH2V1-03	289977	290271	295	-	290272	CACAGTG	22	ACAAAAACC	290309	38	
IGH2V1-02	293835	294126	292	-	294127	CACAGTG	22	ACAAAAACC	294164	38	
IGH2V2-01	303780	304074	295	-	304075	CAGGGCC	24	AGCACAAAG	304114	40	
IGH2V1-01	304926	305204	279	-	305205	CACAGTG	22	TCAAAACCC	305242	38	

Table S9: Co-ordinate table of VH segments in the *N. furzeri IGH* locus

Table S10: Co-ordinate table of DH segments in the *N. furzeri IGH* locus

Name	Start	NT Sequence	End	Length	Strand
IGH1D01	25782	ATACGTACTTTCGTGGTATATAGAGA	25807	26	+
IGH1D02	76700	GATATCTGGGTGGGGG	76715	16	+
IGH1D03	77027	TGAAATGATTAC	77038	12	+
IGH1D04	77476	TCGCGTAGCGGC	77487	12	+
IGH1D05	78717	GAAACCACGGCAGC	78730	14	+
IGH1D06	79049	TTTATAGCGGCTAC	79062	14	+
IGH1D07	80417	CAGACTGGAGA	80427	11	+
IGH1D08	81362	TTCATGGCAGCCAC	81375	14	+
IGH1D09	82067	CAGACTGGAGC	82077	11	+
IGH1D10	84282	TGGGGTGGCAGC	84293	12	+
IGH2D04	263497	CAGACTGGAGA	263507	11	-
IGH2D03	270243	TTTATAGCGGCTAC	270256	14	-
IGH2D02	270878	GAAACCACGGCAGC	270891	14	-
IGH2D01	271749	GACTTTTACTAC	271760	12	-

Table S11: Co-ordinate table of DH 5'-RSSs in the *N. furzeri IGH* locus

Name	5'-RSS Start	Nonamer	Spacer Length	Heptamer	5'-RSS End	Length
IGH1D01	25754	GGTTGTTGT	12	CACTGTG	25781	28
IGH1D02	76672	AGTTTTTGA	12	CACAGTG	76699	28
IGH1D03	76999	TGTTGTTGT	12	CACAGTG	77026	28
IGH1D04	77448	AGTTTTTGT	12	CACGGTG	77475	28
IGH1D05	78688	GATGTTTTT	13	CACAGTG	78716	29
IGH1D06	79021	TGTTTTTGT	12	CGCTGTG	79048	28
IGH1D07	80389	AGTTTTGGT	12	CACAGTG	80416	28
IGH1D08	81334	TGTTTTTGT	12	CGCTGTG	81361	28
IGH1D09	82039	AGTTTTGGT	12	CACAGTG	82066	28
IGH1D10	84254	TCATTCATT	12	CACTGTG	84281	28
IGH2D04	263469	AGTTTTGGT	12	CACAGTG	263496	28
IGH2D03	270215	TGTTTTTGT	12	CGCTGTG	270242	28
IGH2D02	270850	TGTTTTTGT	12	CACAGTG	270877	28
IGH2D01	271721	AGTTTTTAT	12	CATGGTG	271748	28

Table S12: Co-ordinate table of DH 3'-RSSs in the *N. furzeri IGH* locus

Name	3'-RSS Start	Heptamer	Spacer Length	Nonamer	3'-RSS End	Length
IGH1D01	25808	CACAGTG	12	ACAAAAACC	25835	28
IGH1D02	76716	CACAGTG	12	ACAAAAACC	76743	28
IGH1D03	77039	CACTGTG	11	AATATAACC	77065	27
IGH1D04	77488	CACAGCG	12	ACATAAAAC	77515	28
IGH1D05	78731	CACAGCG	12	ACAAAAGCC	78758	28
IGH1D06	79063	CACTGTG	12	ACAAGATCC	79090	28
IGH1D07	80428	CACAACG	12	ACAAAAACC	80455	28
IGH1D08	81376	CACTGTG	12	ACAAAATCC	81403	28
IGH1D09	82078	CACAATG	12	ACAAAAACC	82105	28
IGH1D10	84294	CACAGTG	12	ACAAAAACC	84321	28
IGH2D04	263508	CACAACG	12	ACAAAAACC	263535	28
IGH2D03	270257	CACTGTG	12	ACAAGATCC	270284	28
IGH2D02	270892	CACAGCG	12	ACAAAAGCC	270919	28
IGH2D01	271761	CACAATG	12	ACAAAAACC	271788	28

Name	Start	NT Sequence	AA Sequence	End	Length	Strand
IGH1J01	26187	GTGCTTTAGACAACTGGGGAAAAGGAACGGAGGTTACTGTTCAACCTG	ALDNWGKGTEVTVQP	26234	48	+
IGH1J02	128176	ATGACTACTTTGACTACTGGGGAAAAGGAACAATGGTGACGGTCACATCAG	DYFDYWGKGTMVTVTS	128226	51	+
IGH1J03	128354	ACCGTGGGGTAAAGGGACAACAGTCACGGTCAAAACAG	PWGKGTTVTVKT	128391	38	+
IGH1J04	128533	ACGGTGCTCTTGACTACTGGGGTAAAGGGACCGCAGTCACTGTAACATCAG	GALDYWGKGTAVTVTS	128583	51	+
IGH1J05	128887	ACAACGCTTTTGACTACTGGGGAAAAGGAACAACGGTCACCGTCACTTCAG	NAFDYWGKGTTVTVTS	128937	51	+
IGH1J06	129346	CTACGATGCTTTTGACTACTGGGGGAAAAGGACGATGGTCACGTCACTTCAG	YDAFDYWGKRTMVTSLQ	129397	52	+
IGH1J07	129635	TTAACTGGGCTTTCGACTACTGGGGAAAAGGGACGATGGTAACGGTGACTTCAG	NWAFDYWGKGTMVTVTS	129688	54	+
IGH1J08	129965	TTACCACGCAGCTTTGGACTACTGGGGAAAAGGGACGACGGTCACCGTCACCTCAG	YHXALDYWGKGTTVTVTS	130020	56	+
IGH1J09	130612	TCTACGCTGCTTTTGACTACTGGGGTAAAGGTACAACGGTAACCGTTTCATCAG	YAAFDYWGKGTTVTVSS	130665	54	+
IGH2J08	204031	TCTACGCTGCTTTTGACTACTGGGGTAAAGGTACAACGGTAACCGTTTCATCAG	YAAFDYWGKGTTVTVSS	204084	54	-
IGH2J07	204673	TTACCACGCAGCTTTGGACTACTGGGGAAAAGGGACGACGGTCACCGTCACCTCAG	YHXALDYWGKGTTVTVTS	204728	56	-
IGH2J06	205005	ATAACTGGGCTTTCGACTACTGGGGAAAAGGGACGATGGTAACGGTGACTTCAG	NWAFDYWGKGTMVTVTS	205058	54	-
IGH2J05	205296	CTACGATGCTTTTGACTACTGGGGGAAAAGGACGATGGTCACGTCACTTCAG	YDAFDYWGKRTMVTSLQ	205347	52	-
IGH2J04	205756	ACAACGCTTTTGACTACTGGGGAAAAGGAACAACGGTCACCGTCACTTCAG	NAFDYWGKGTTVTVTS	205806	51	-
IGH2J03	206111	ATGGTGCTTTTGACTACTGGGGTAAAGGGACCGCAGTCACTGTAACATCAG	GAFDYWGKGTAVTVTS	206161	51	-
IGH2J02	206303	ACCGTGGGGTAAAGGGACAACAGTCACGGTCAAAACAG	PWGKGTTVTVKT	206340	38	-
IGH2J01	206466	ATGACTACTTTGACTACTGGGGAAAAGGAACAATGGTGACGGTCACATCAG	DYFDYWGKGTMVTVTS	206516	51	-

Table S13: Co-ordinate table of JH segments in the *N. furzeri IGH* locus

Name	RSS Start	Nonamer	Spacer Length	Heptamer	RSS End	RSS Length
IGH1J01	26196	TGTTTTTGT	23	CACTGTG	26186	39
IGH1J02	128188	AGTGTTTGT	23	CACTGTG	128175	39
IGH1J03	128353	TGTTTATTT	23	CACTGTG	128353	39
IGH1J04	128545	GGTTTTTGT	23	CACTGTG	128532	39
IGH1J05	128899	GGTTTTAGT	23	TACTGTG	128886	39
IGH1J06	129360	TCTTCTTGT	22	TACTTTG	129345	38
IGH1J07	129650	AGTTTTTGT	23	TACTGTG	129634	39
IGH1J08	129983	AGTTTTAGT	22	TACTGTG	129964	38
IGH1J09	130628	CGTTTTTAT	22	CACTGTG	130611	38
IGH2J08	204047	CGTTTTTAT	22	CACTGTG	204030	38
IGH2J07	204691	AGTTTTAGT	22	TACTGTG	204672	38
IGH2J06	205020	AGTTTTTGT	23	TACTGTG	205004	39
IGH2J05	205310	TCTTCTTGT	22	TACTTTG	205295	38
IGH2J04	205768	GGTTTTAGT	23	TACTGTG	205755	39
IGH2J03	206123	GGTTTTTGT	23	CACTGTG	206110	39
IGH2J02	206302	TGTTTATTT	23	CACTGTG	206302	39
IGH2J01	206478	AGTGTTTGT	23	CACTGTG	206465	39

Table S14: Co-ordinate table of JH RSSs in the *N. furzeri IGH* locus

Table S15: Co-ordinate table of constant-region exons in the *X. maculatus IGH* locus

Name	Isotype	Start	End	Length	Strand
IGHZ1-1	Z	3380	3667	288	+
IGHZ1-2	Z	3814	4098	285	+
IGHZ1-3	Z	4195	4497	303	+
IGHZ1-4	Z	4934	5263	330	+
IGHZ1-S	Z	5264	5459	196	+
IGHZ1-TM1	Z	6345	6490	146	+
IGHZ1-TM2	Z	6645	7043	399	+
IGHZ2-1	Z	256059	256337	279	+
IGHZ2-2	Z	256453	256734	282	+
IGHZ2-3	Z	256893	257171	279	+
IGHZ2-4	Z	257319	257636	318	+
IGHZ2-S	Z	257637	257850	214	+
IGHZ2-TM1	Z	258059	258213	155	+
IGHZ2-TM2	Z	258410	258629	220	+
IGHM-1	M	279664	279960	297	+
IGHM-2	M	280880	281224	345	+
IGHM-3	M	281321	281629	309	+
IGHM-4	M	281789	282291	503	+
IGHM-TM1	M	282910	283034	125	+
IGHM-TM2	M	285028	285740	713	+
IGHD-1	D	285902	286219	318	+
IGHD-2A	D	286310	286597	288	+
IGHD-3A	D	286814	287128	315	+
IGHD-4A	D	287250	287534	285	+
IGHD-2B	D	288876	289166	291	+
IGHD-3B	D	289262	289576	315	+
IGHD-4B	D	289680	289964	285	+
IGHD-5	D	290052	290381	330	+
IGHD-6	D	290472	290789	318	+
IGHD-7	D	290865	291152	288	+
IGHD-TM1	D	291286	291434	149	+
IGHD-TM2	D	291541	291642	102	+

Name	Start	End	Length	Strand	RSS Start	Heptamer	Spacer Length	Nonamer	RSS End	RSS Length	Comment
IGHV01-01	1159	1450	292	+	1451	CACAGTG	23	GTAAAAACC	1489	39	
IGHV02-01	10534	10825	292	+	10826	CACAGTG	23	ACAAAACCC	10864	39	
IGHV02-02	11961	12261	301	+	12262	CACTGTG	23	ACAAAAACT	12300	39	
IGHV02-03	13319	13616	298	+	13617	CACAGTG	23	ACACAAACT	13655	39	
IGHV03-01	15440	15734	295	+	15735	CACAGTG	22	ACAAAAACT	15772	38	
IGHV02-04	16618	16908	291	+	16909	CACAGTG	23	ACAAAAACC	16947	39	
IGHV02-05	17522	17822	301	+	17823	CACTGTG	22	ACAAAAACT	17860	38	
IGHV02-06	18881	19178	298	+	19179	CACAGTG	23	ACACAAACT	19217	39	
IGHV03-02	21000	21294	295	+	21295	CACAGTG	22	ACAAAAACT	21332	38	
IGHV02-07	22179	22467	289	+	22468	CACAGTG	23	ACAAAAACC	22506	39	
IGHV02-08p	24234	24514	281	+	24515	CACAGTG	23	ACAAAAACT	24553	39	Frameshift
IGHV04-01	25359	25659	301	+	25660	CACAGTG	23	ACAAAAACT	25698	39	
IGHV04-02	27066	27366	301	+	27367	CACAGTG	23	ACAAAAACA	27405	39	
IGHV02-09	28669	28958	290	+	28959	CACAGTG	23	ACAAAAACC	28997	39	
IGHV02-10p	30460	30741	282	+	30742	CACAATG	23	ACAAAACTC	30780	39	Frameshift
IGHV02-11	32395	32681	287	+	32682	CACAGTG	23	ACAAAAACC	32720	39	
IGHV03-03	33663	33957	295	+	33958	CACTGTG	22	ACAAAAACT	33995	38	
IGHV02-12	35012	35299	288	+	35300	CACAGTG	23	ACAAAAACC	35338	39	
IGHV03-04	36281	36575	295	+	36576	CACTGTG	22	ACAAAAACT	36613	38	
IGHV02-13	37639	37931	293	+	37932	CACAGTG	23	ACAAAAACT	37970	39	
IGHV02-14	39019	39311	293	+	39312	CACAGTG	23	ACAAAAACT	39350	39	
IGHV03-05	41008	41302	295	+	41303	CACAGTG	22	ACAAAAACT	41340	38	
IGHV02-15	42660	42952	293	+	42953	CACAGTG	23	ACAAAAACT	42991	39	
IGHV03-06	45081	45375	295	+	45376	CACAGTG	22	ACAAAAACT	45413	38	
IGHV02-16	46732	47024	293	+	47025	CACAGTG	23	ACAAAAACT	47063	39	

Table S16: Co-ordinate table of VH segments in the *X. maculatus IGH* locus, part 1

Name	Start	End	Length	Strand	RSS Start	Heptamer	Spacer Length	Nonamer	RSS End	RSS Length	Comment
IGHV03-07	48618	48912	295	+	48913	CACAGTG	22	ACAAAAACT	48950	38	
IGHV02-17	50323	50611	289	+	50612	CACAGTG	23	ACAAAAACC	50650	39	
IGHV03-08	51890	52184	295	+	52185	CACAGTG	22	ACAAAAACT	52222	38	
IGHV03-09p	53026	53274	249	+	53275						3'-truncated, no RSS
IGHV02-18	54462	54747	286	+	54748	CACAGTG	23	ACAAAAACC	54786	39	
IGHV02-19p	55729	55866	138	+	55867	CACAGTG	23	ACAAAAACC	55905	39	3'-truncated
IGHV03-10	57371	57662	292	+	57663	CACAGTG	22	ACAAAAACT	57700	38	
IGHV02-20p	58698	58986	289	+	58987	CACAGTG	23	ATAAAAACC	59025	39	Nonsense mutation
IGHV03-11	59940	60234	295	+	60235	CACAGTG	22	ACAAAAACT	60272	38	
IGHV02-21	61249	61537	289	+	61538	CACAGTG	23	ATAAAAACC	61576	39	
IGHV03-12	62491	62785	295	+	62786	CACAGTG	22	ACAAAAACT	62823	38	
IGHV02-22	63801	64089	289	+	64090	CACAGTG	23	ATAAAAACC	64128	39	
IGHV03-13	65043	65337	295	+	65338	CACAGTG	22	ACAAAAACT	65375	38	
IGHV02-23	66354	66640	287	+	66641	CACAGTG	23	ACAAAAACT	66679	39	
IGHV03-14	68452	68743	292	+	68744	CACTATG	22	ACAAAACTC	68781	38	
IGHV02-24	70101	70389	289	+	70390	CACAGTG	23	ACAAAAACC	70428	39	
IGHV03-15	72206	72501	296	+	72502	CACAGTG	22	ACAAAAACT	72539	38	
IGHV02-25	73484	73772	289	+	73773	CACAGTG	23	ACAAAAACC	73811	39	
IGHV03-16	75799	76090	292	+	76091	CACAGTG	22	ACAAAAACT	76128	38	
IGHV03-17	77773	78067	295	+	78068	CACAGTG	22	ACAAAAACT	78105	38	
IGHV02-26	79001	79289	289	+	79290	CACAGTG	23	ACAAAAACC	79328	39	
IGHV03-18	80492	80784	293	+	80785	CACAGTG	22	ACAAAAACT	80822	38	
IGHV02-27p	81799	82082	284	+	82083	CACAGTG	23	ACAAAAACC	82121	39	Frameshift
IGHV03-19	83736	84030	295	+	84031	CACAGTG	22	ACAAAAACT	84068	38	
IGHV02-28p	85093	85381	289	+	85382	CACAGGG	23	GCAAAAACC	85420	39	Nonsense mutation

Table S17: Co-ordinate table of VH segments in the *X. maculatus IGH* locus, part 2

Name	Start	End	Length	Strand	RSS Start	Heptamer	Spacer Length	Nonamer	RSS End	RSS Length	Comment
IGHV02-29	86225	86505	281	+	86506	CACAGTG	23	ATAAAAACC	86544	39	
IGHV03-20	87419	87713	295	+	87714	CACAGTG	22	ACAAAAACT	87751	38	
IGHV03-21	94532	94826	295	+	94827	CACAGTG	23	ACAAAAACC	94865	39	
IGHV03-22	96192	96489	298	+	96490	CACAGTG	23	ACAAAAACC	96528	39	
IGHV03-23	98068	98368	301	+	98369	CACAGTG	23	ACAAAAACC	98407	39	
IGHV03-24	99482	99779	298	+	99780	CACAGTG	23	ACAAAAACC	99818	39	
IGHV03-25	101639	101936	298	+	101937	CACAGTG	23	ACAAAAACC	101975	39	
IGHV05-01p	102818	103096	279	+	103097	CAGAAGC	0	ACAAAAACT	103112	16	Frameshift
IGHV03-26	104098	104389	292	+	104390	CACAGTG	23	ACAAAATCC	104428	39	
IGHV06-01	105551	105831	281	+	105832	CACAGTG	23	ACAAAAACC	105870	39	
IGHV03-27	107274	107571	298	+	107572	CACAGTG	23	ACAAAAACC	107610	39	
IGHV03-28	108775	109072	298	+	109073	CACAGAG	23	ACAAAAACC	109111	39	
IGHV03-29	110372	110672	301	+	110673	CACAGTG	23	ACAAAAACC	110711	39	
IGHV07-01	111565	111856	292	+	111857	CACAATG	23	ACAAAAACT	111895	39	
IGHV08-01p	113033	113330	298	+	113331	CACAGAG	23	CCAAGAACC	113369	39	Nonsense mutation
IGHV09-01	115512	115800	289	+	115801	CACAGTG	22	ACAAAAACT	115838	38	
IGHV10-01	117078	117379	302	+	117380	CACAGTG	22	ACATAAACT	117417	38	
IGHV11-01	119462	119760	299	+	119761	CACAGTG	23	ACAAAAACT	119799	39	
IGHV03-30	126125	126416	292	+	126417	CACAGTG	22	ACAAAAACC	126454	38	
IGHV03-31	127109	127400	292	+	127401	CACAGTG	23	GCAAAAACC	127439	39	
IGHV12-01	128489	128786	298	+	128787	CACAGTG	23	ACAAAAACC	128825	39	
IGHV02-30	135711	136000	290	+	136001	CACAGTG	22	ACAAAAACA	136038	38	
IGHV13-01	136757	137057	301	+	137058	CACAGTG	23	ACAAAAACT	137096	39	
IGHV02-31	138344	138637	294	+	138638	CACAGTG	23	ACAAAAATC	138676	39	
IGHV02-32	140024	140315	292	+	140316	CACTGTG	23	ACAAAAACT	140354	39	

Table S18: Co-ordinate table of VH segments in the *X. maculatus IGH* locus, part 3

Name	Start	End	Length	Strand	RSS Start	Heptamer	Spacer Length	Nonamer	RSS End	RSS Length	Comment
IGHV02-33	142332	142620	289	+	142621	CACAGTG	23	ACAAAAACA	142659	39	
IGHV02-34	144334	144625	292	+	144626	CACAGTG	23	ACAAAAACT	144664	39	
IGHV02-35	145740	146031	292	+	146032	CACAGTG	23	ACAAAAAAT	146070	39	
IGHV02-36	146903	147194	292	+	147195	CACAGTG	23	ACAAAAACT	147233	39	
IGHV02-37	147839	148138	300	+	148139	CACAGTG	23	ACAAAAATC	148177	39	
IGHV02-38p	150504	150797	294	+	150798	CACAATA	23	ACAAAAACC	150836	39	Nonsense mutation
IGHV02-39	152249	152537	289	+	152538	CACAGTA	23	ACAAAAACC	152576	39	
IGHV14-01	154075	154374	300	+	154375	CACAGTG	23	ACAAAAAGT	154413	39	
IGHV02-40	155433	155709	277	+	155710	CACAGTG	23	ACAAAAACC	155748	39	
IGHV02-41	156583	156870	288	+	156871	CACAGTG	23	ACAAAAACC	156909	39	
IGHV02-42	163977	164269	293	+	164270	CACAGTG	23	ACAAAACCC	164308	39	
IGHV03-32	165416	165708	293	+	165709	CACAGTG	22	ACAAAAACA	165746	38	
IGHV02-43	166994	167293	300	+	167294	CACAATG	23	ACAGAAACT	167332	39	
IGHV12-02	169602	169900	299	+	169901	CACAGTG	23	ACAAAAACC	169939	39	
IGHV02-44	171452	171752	301	+	171753	CACTGTG	23	GCAAAAACT	171791	39	
IGHV02-45	173096	173384	289	+	173385	CTCAGTG	23	ACAAAAACC	173423	39	
IGHV02-46	174714	175009	296	+	175010	CACAGTG	23	ACAAAAACT	175048	39	
IGHV02-47	176396	176697	302	+	176698	CACAGTG	23	ACAAAAACT	176736	39	
IGHV12-03	178422	178719	298	+	178720	CACAGTG	23	ACAAAAACA	178758	39	
IGHV12-04	181245	181543	299	+	181544	CACAGTG	23	ACAAAAACC	181582	39	
IGHV02-48p	182977	183236	260	+	183237	CACAGGT	8	ACAAAAACT	183260	24	5'-truncated
IGHV02-49p	184323	184611	289	+	184612	CACAGTG	23	ACAAAAACC	184650	39	Nonsense mutation
IGHV02-50	185946	186244	299	+	186245	CACAGTG	23	ACAAAAACT	186283	39	
IGHV02-51	187624	187925	302	+	187926	CACAGTG	23	ACAAAAACT	187964	39	
IGHV12-05	190987	191284	298	+	191285	CACAGTG	23	ACAAAAACA	191323	39	

Table S19: Co-ordinate table of VH segments in the *X. maculatus IGH* locus, part 4

Name	Start	End	Length	Strand	RSS Start	Heptamer	Spacer Length	Nonamer	RSS End	RSS Length	Comment
IGHV02-52	192570	192868	299	+	192869	CACAGTG	19	CTGAAAACC	192903	35	
IGHV12-06	193608	193906	299	+	193907	CACAGTG	23	ACAAAAACA	193945	39	
IGHV02-53	195271	195572	302	+	195573	CACAGTG	23	ACAAAAACC	195611	39	
IGHV15-01	204396	204693	298	+	204694	CACAATC	23	ACAAAAACT	204732	39	
IGHV13-02	206203	206503	301	+	206504	CACAGTG	23	ACAAAAACT	206542	39	
IGHV16-01	207726	208020	295	+	208021	CACAGTG	22	ACAAAAACT	208058	38	
IGHV13-03	208477	208777	301	+	208778	CACAGTA	23	ACAAAAACT	208816	39	
IGHV03-33	209921	210215	295	+	210216	CACGGTG	22	ACGAAAACT	210253	38	
IGHV17-01	211322	211625	304	+	211626	CACAGTA	23	ACAAAAACC	211664	39	
IGHV15-02p	214600	214860	261	+	214861						3'-truncated, no RSS
IGHV18-01	215671	215962	292	+	215963	CACACTG	23	ACAAAAACC	216001	39	
IGHV19-01	217874	218174	301	+	218175	CACAGTG	23	ACAAAAACT	218213	39	
IGHV03-34	219368	219668	301	+	219669	CACAGTG	23	ACAAAAACA	219707	39	
IGHV20-01	220329	220632	304	+	220633	CACAGTG	23	ACAAAAATT	220671	39	
IGHV02-54p	228547	228838	292	+	228839	CACACTG	23	ACAACCCCC	228877	39	Nonsense mutation
IGHV02-55	229963	230267	305	+	230268	CACAGCG	23	ACAAAAAAA	230306	39	
IGHV03-35	231630	231928	299	+	231929	CACAGTG	23	ACAAAAACC	231967	39	
IGHV21-01p	233069	233230	162	+	233231						Nonsense mutation, 3'-truncated, no RSS
IGHV22-01p	234954	235102	149	+	235103	CACAGTG	23	TCAAAAACT	235141	39	5'-truncated
IGHV02-56	236029	236330	302	+	236331	CACAGTG	23	ACAAATACT	236369	39	
IGHV03-36p	238122	238413	292	+	238414	CACAATG	23	ACAGAATCC	238452	39	Nonsense mutation
IGHV11-02p	240281	240579	299	+	240580	CACAGTG	24	ACAAAAACT	240619	40	Nonsense mutation
IGHV09-02	241878	242166	289	+	242167	CACAGTG	22	ACAAAAACT	242204	38	
IGHV23-01	243867	244164	298	+	244165	CACAGTG	23	ACAAAATCC	244203	39	
IGHV02-57	245524	245813	290	+	245814	CACCATA	22	ACAAAATCC	245851	38	

Table S20: Co-ordinate table of VH segments in the *X. maculatus IGH* locus, part 5

Table S21: Co-ordinate table of DH segments in the *X. maculatus IGH* locus

Name	Start	NT Sequence	End	Length	Strand
IGHDZ01	2243	GTGGGCAGGAGGCTATGC	2260	18	+
IGHDZ02	119768	AGG	119770	3	+
IGHDZ03	128794	ACTAAAGG	128801	8	+
IGHDZ04	129907	ATCGGG	129912	6	+
IGHDZ05	158017	ATATATGGGGG	158027	11	+
IGHDZ06	197791	ATATACTGGGGTGG	197804	14	+
IGHDZ07	222022	ATGGACTGGGGGG	222034	13	+
IGHDZ08	247941	GTGATTACGGCTACGGGGC	247959	19	+
IGHDZ09	249514	TTATGGGCTGGGGAG	249528	15	+
IGHDZ10	253752	TGGGTGGGGC	253761	10	+
IGHDM01	267392	TATACAGTGGCAAC	267405	14	+
IGHDM02	268498	CAGTATAGCAAC	268509	12	+
IGHDM03	268836	TACAATGGCAAC	268847	12	+
IGHDM04	269694	TAAACAGTGGCTAC	269707	14	+

Table S22: Co-ordinate table of DH 5'-RSSs in the *X. maculatus IGH* locus

Name	5'-RSS Start	Nonamer	Spacer Length	Heptamer	5'-RSS End	Length
IGHDZ01	2215	GGTTTTTGT	12	CACTGTG	2242	28
IGHDZ02	119739	TGTATTACT	13	CACAGTG	119767	29
IGHDZ03	128766	TTTACTTCT	12	CACAGTG	128793	28
IGHDZ04	129879	GGTTTTTGT	12	CACAGTG	129906	28
IGHDZ05	157989	AGTTTTTGT	12	CACAGTG	158016	28
IGHDZ06	197763	GGTTTTTGC	12	TACTGTG	197790	28
IGHDZ07	221994	GGTTTTTGT	12	CGCTGTG	222021	28
IGHDZ08	247913	TGTTTTTGT	12	ATCTGTG	247940	28
IGHDZ09	249486	AGTTTTTGT	12	TGTGGTG	249513	28
IGHDZ10	253724	AGTTTTTGT	12	TGTAGTG	253751	28
IGHDM01	267364	AGTTTTTGT	12	TACAGTG	267391	28
IGHDM02	268470	TGTTTTTGT	12	CACAGTG	268497	28
IGHDM03	268808	AGTTTTTGC	12	TACTGTG	268835	28
IGHDM04	269666	CGTTTTTGT	12	CATTGTG	269693	28

Table S23: Co-ordinate table of DH 3'-RSSs in the *X. maculatus IGH* locus

Name	3'-RSS Start	Heptamer	Spacer Length	Nonamer	3'-RSS End	Length
IGHDZ01	2261	CACTAAG	12	ACAAAAAGT	2288	28
IGHDZ02	119771	CAAAATG	13	ACAAAAACT	119799	29
IGHDZ03	128802	CAGAGAA	8	ACAAAAACC	128825	24
IGHDZ04	129913	CACAATG	12	TCAAAAACC	129940	28
IGHDZ05	158028	CACAGAG	12	ACAAAAACC	158055	28
IGHDZ06	197805	CACACAG	12	ACAAAAACC	197832	28
IGHDZ07	222035	CACAGAG	12	ACAAAAACC	222062	28
IGHDZ08	247960	CACAATA	12	ACAAAAACC	247987	28
IGHDZ09	249529	CACAATG	12	ACAAAAACC	249556	28
IGHDZ10	253762	CACAGTA	12	ACAAAAACC	253789	28
IGHDM01	267406	CACAGTG	12	GCAAAAACC	267433	28
IGHDM02	268510	CACAGTG	12	ACAGAAACC	268537	28
IGHDM03	268848	CACAGTG	12	ACAAAAACC	268875	28
IGHDM04	269708	CACTGTG	12	ACAAAATCA	269735	28

Name	Start	NT Sequence	AA Sequence	End	Length	Strand
IGHJZ01	2653	ATGCCTTAGATTACTGGGGTGAAGGGACCAGAGTCACAGTGACTTCAG	ALDYWGEGTRVTVTS	2700	48	+
IGHJZ02	120639	ATTACGCTCTTGACTACTGGGGAGCAGGAACCAAAGTTACTGTAAAGCCAG	YALDYWGAGTKVTVKP	120689	51	+
IGHJZ03	130376	ACTACGGCTTTGATTACTGGGGAGACGGAACTGAAGTTACTGTTGAACCAG	YGFDYWGDGTEVTVEP	130426	51	+
IGHJZ04	158408	AGATTTAGACTACTGGGGTAATGGAACAACAGTCACGGTTCTACCAG	DLDYWGNGTTVTVLP	158454	47	+
IGHJZ05	198186	ATTATGGTTTTGACTACTGGGGAGACGGAACCACAGTCACTGTTAGTCCAG	YGFDYWGDGTTVTVSP	198236	51	+
IGHJZ06	222417	ATGCTTTTGACGTCTGGGGTAAAGGAACCACAGTTACTGTTGTACCAG	AFDVWGKGTTVTVVP	222464	48	+
IGHJZ07	254130	ATGTTTTTGACTACTGGGGTAAAGGGACTGATGTCACAGTATCTCCAG	VFDYWGKGTDVTVSP	254177	48	+
IGHJM01	276014	ACGGCTACTTCGACTACTGGGGGAAAGGAACACAAGTCACAGTGACTTCTG	GYFDYWGKGTQVTVTS	276064	51	+
IGHJM02	276284	CCACTACTTTGACTACTGGGGAAAAGGAACCACGGTTACCGTCACTTCAG	HYFDYWGKGTTVTVTS	276333	50	+
IGHJM03	276654	ACAATGCTTTTGACTACTGGGGAAAAGGAACTACGGTAACAGTAACATCAG	NAFDYWGKGTTVTVTS	276704	51	+
IGHJM04	276999	ACTACGCTTTTGACTACTGGGGAAAAGGAACAATGGTCACTGTCACTTCAG	YAFDYWGKGTMVTVTS	277049	51	+
IGHJM05	277322	ACAACTGGGCTTTTGACTACTGGGGAGCAGGAACCATGGTAACAGTAACATCAG	NWAFDYWGAGTMVTVTS	277375	54	+
IGHJM06	277672	CTACGGTGCTTTTGACTACTGGGGTAAAGGGACTACAGTCACCGTCACTTCAG	YGAFDYWGKGTTVTVTS	277724	53	+
IGHJM07	278150	CTACGATGCTTTTGACTATTGGGGGAAAGGAACAACAGTCACCGTCATCACTTCAG	YDAFDYWGKGTTVTVITS	278205	56	+
IGHJM08	278606	TTACTACTACGCTTTTGACTATTGGGGAAAAGGGACAATGGTCACCGTCACTTCAG	YYYAFDYWGKGTMVTVTS	278661	56	+

Table S24: Co-ordinate table of JH segments in the *X. maculatus IGH* locus

Name	RSS Start	Nonamer	Spacer Length	Heptamer	RSS End	RSS Length
IGHJZ01	2662	TGTTTTTGT	23	CACTGTG	2652	39
IGHJZ02	120651	TGTTTTTGT	23	CACTGTG	120638	39
IGHJZ03	130388	TGTTTTTGT	23	CACCGTG	130375	39
IGHJZ04	158416	GGTTTTTGT	23	CACTGTG	158407	39
IGHJZ05	198198	GGTTTTTGT	23	CACTGTG	198185	39
IGHJZ06	222426	TGTTTTTGT	23	CACTGTG	222416	39
IGHJZ07	254139	GGTTTTTGT	23	CACTGTG	254129	39
IGHJM01	276026	TGTATTTGT	23	CACTGTG	276013	39
IGHJM02	276295	TATTTTTGC	23	CACCGTG	276283	39
IGHJM03	276666	TGTTTTTGT	23	TACTGTG	276653	39
IGHJM04	277011	TGTTTTAGT	23	TACTGTG	276998	39
IGHJM05	277338	GGTTTTTGT	22	TACTGTG	277321	38
IGHJM06	277687	GCTTTTTAT	22	CACTGTG	277671	38
IGHJM07	278168	CCTTTTTAC	22	CACTGTG	278149	38
IGHJM08	278624	GCTTTTTAA	22	CACTGTG	278605	38

Table S25: Co-ordinate table of JH RSSs in the *X. maculatus IGH* locus

Species	Scaffold(s)	Region	Isotype	Known Exons [1]	Complete?	Pseudo-exons	Comments
Nothobranchius orthonotus	scf33878	IGHM1	M	1,2,3,TM1	**No**	–	CM4 missing (missing sequence)
Nothobranchius orthonotus	scf33878	IGHD1	D	1,2,3,4,2,3,4,5,6,7,TM1	Yes	–	
Nothobranchius orthonotus	scf34438	IGHM2	M	1,2,3,4,TM1	Yes	–	
Nothobranchius orthonotus	scf34438, scf33917	IGHD2	D	1,2,3,4,2,3,4,5,6,7,TM1	Yes	–	
Nothobranchius orthonotus	scf33917	IGHD3	D	1,2,3,4,2,3,4,5,6,7,TM1	Yes	–	
Nothobranchius orthonotus	scf33917	IGHD4	D	1,2,3,4,2,3,4,5,6,7,TM1	Yes	–	
Nothobranchius orthonotus	scf9255, scf26119, scf33917	IGHD5	D	3,4,2,3,4,5,6,7,TM1	**No**	–	CD1 & CD2A missing (missing sequence)
Nothobranchius orthonotus	scf27951, scf33789	IGHM3	M	1,2,3,4,TM1	Yes	–	
Nothobranchius orthonotus	scf27951, 32033	IGHD6	D	1,2,3,4,2,3,4,5,6,7,TM1	Yes	–	
Nothobranchius orthonotus	scf32137, scf21286	IGHM4	M	1,2,3,4,TM1	Yes	–	
Nothobranchius furzeri	chr6 + BACs	IGH1M	M	1,2,3,4,TM1	Yes	–	
Nothobranchius furzeri	chr6 + BACs	IGH1D	D	1,2,3,4,2,3,4,5,6,7,TM1	Yes	–	
Nothobranchius furzeri	chr6 + BACs	IGH2M	M	1,2,3,4,TM1	Yes	–	
Nothobranchius furzeri	chr6 + BACs	IGH2D	D	1,2,3,4,2,3,4,5,6,7,TM1	Yes	–	
Aphyosemion australe	scf373	IGHM	M	1,2,3,4,TM1	Yes	–	
Aphyosemion australe	scf373	IGHD	D	1,2,3,4,5,6,7,TM1	Yes	–	
Callopanchax toddi	scf107	IGHZ1	Z	1,2,3,4,TM1	Yes	–	
Callopanchax toddi	scf107	IGHZ2	Z	1,2,3,4,TM1	Yes	–	
Callopanchax toddi	scf1209	IGHZ3	Z	1,2,3,4,TM1	Yes	–	
Callopanchax toddi	scf1209	IGHM1	M	1	**No**	–	Isolated CM1 exon
Callopanchax toddi	scf945	IGHZ4	Z	1,2,3,4,TM1	Yes	–	
Callopanchax toddi	scf945	IGHM2	M	1,2,3,4,TM1	Yes	–	
Callopanchax toddi	scf945	IGHD1	D	1,2,3,4,5,6,7,TM1	Yes	1,4,5	Frameshift mutations in CD1, CD4 & CD5
Callopanchax toddi	scf265	IGHM3	M	1,2,3,4,TM1	Yes	–	
Callopanchax toddi	scf265	IGHD2	D	1,5,7,TM1	**No**	–	CD2-4 & CD5-6 missing (not in sequence)

[1] Excluding TM2 and secretory exons.

Table S26: *IGH* constant regions in cyprinidontiform fish, part 1

**Species**	**Scaffold(s)**	**Region**	**Isotype**	**Known Exons** [1]	**Complete?**	**Pseudo-exons**	**Comments**
Pachypanchax playfairii	scf547	IGHZ	Z	1,2,3,4,TM1	Yes	–	
Pachypanchax playfairii	scf125	IGHM1	M	1,2,3,4,TM1	Yes	–	
Pachypanchax playfairii	scf125	IGHD	D	1,2,3,4,5,6,7,TM1	Yes	–	
Pachypanchax playfairii	scf547	IGHM2	M	1	**No**	–	Isolated CM1 exon
Austrofundulus limnaeus	NW_013954375.1	IGHZ	Z	TM1	**No**	TM1	Isolated TM1 exon with frameshift mutation
Austrofundulus limnaeus	NW_013952673.1	IGHM	M	1,2,3,4,TM1	Yes	–	
Austrofundulus limnaeus	NW_013952673.1, NW_013956335.1	IGHD	D	1,2,3,4,5,6,7,TM1	Yes	–	
Kryptolebias marmoratus	NW_016094348.1	IGHZ1	Z	1,2,3,4,TM1	Yes	–	
Kryptolebias marmoratus	NW_016094348.1	IGHZ2	Z	1,4,TM1	**No**	–	CZ2 & CZ3 missing (not in sequence)
Kryptolebias marmoratus	NW_016094301.1	IGHM1	M	1,2,3,4,TM1	Yes	–	
Kryptolebias marmoratus	NW_016094301.1	IGHD1	D	1,2,3,4,5,6,7,TM1	Yes	–	
Kryptolebias marmoratus	NW_016094277.1	IGHM2	M	1,2,3,4,TM1	Yes	–	
Kryptolebias marmoratus	NW_016094277.1	IGHD2	D	1,2,3,4,5,6,TM1	**No**	–	CD7 missing (not in sequence)
Poecilia reticulata	NC_024338.1	IGHZ1	Z	1,2,3,4	**No**	–	TM1 missing (missing sequence)
Poecilia reticulata	NC_024338.1	IGHZ2	Z	1,2,3,4,TM1	Yes	–	
Poecilia reticulata	NC_024338.1	IGHM	M	1,2,3,4,TM1	Yes	–	
Poecilia reticulata	NC_024338.1	IGHD	D	1,2,3,4,2,3,4,5,6,7,TM1	Yes	–	
Poecilia formosa	NW_006800081.1	IGHZ1	Z	1,2,3,4,TM1	Yes	–	
Poecilia formosa	NW_006800081.1	IGHZ2	Z	1,2,3,4,TM1	Yes	–	
Poecilia formosa	NW_006800081.1	IGHZ3	Z	1,2,3,4,TM1	Yes	–	
Poecilia formosa	NW_006800081.1	IGHM	M	1,2,3,4,TM1	Yes	–	
Poecilia formosa	NW_006800081.1	IGHD	D	1,2,3,4,5,6,7,TM1	Yes	–	
Xiphophorus maculatus	NC_036458	IGHZ1	Z	1,2,3,4,TM1	Yes	–	
Xiphophorus maculatus	NC_036458	IGHZ2	Z	1,2,3,4,TM1	Yes	–	
Xiphophorus maculatus	NC_036458	IGHM	M	1,2,3,4,TM1	Yes	–	

[1] Excluding TM2 and secretory exons.

Table S27: *IGH* constant regions in cyprinidontiform fish, part 2

Species	Scaffold(s)	Region	Isotype	Known Exons [1]	Complete?	Pseudo-exons	Comments
Xiphophorus maculatus	NC_036458	IGHD	D	1,2,3,4,2,3,4,5,6,7,TM1	Yes	–	
Fundulus heteroclitus	NW_012234561.1	IGHZ1	Z	1,2,3,4,TM1	Yes	–	
Fundulus heteroclitus	NW_012230737.1	IGHZ2	Z	4,TM1	**No**	–	CZ1 to CZ3 missing (missing sequence)
Fundulus heteroclitus	NW_012234542.1	IGHM	M	1,2,3,4,TM1	Yes	–	
Fundulus heteroclitus	NW_012234542.1	IGHD	D	1,2,3,4,2,3,4,5,6,7,TM1	Yes	–	
Cyprinodon variegatus	NW_015154250.1, NW_015151047.1	IGHZ	Z	1,2,3,4,TM1	Yes	–	
Cyprinodon variegatus	NW_015151047.1	IGHM	M	1,2,3,4,TM1	Yes	–	
Cyprinodon variegatus	NW_015151047.1	IGHD	D	1,2,3,4,2,3,4,5,6,7,TM1	Yes	–	
Oryzias latipes	NC_019866.2	IGHM1	M	1,2,3,4,TM1	Yes	–	
Oryzias latipes	NC_019866.2	IGHD1	D	1,2,3,4,6,7,TM1	Yes	7	Nonsense mutation in CD7
Oryzias latipes	NC_019866.2	IGHM2	M	1,2,3,4,TM1	Yes	–	
Oryzias latipes	NC_019866.2	IGHD2	D	1,2,3,4,6,7,TM1	Yes	–	
Oryzias latipes	NC_019866.2	IGHM3	M	1,2,3,4,TM1	Yes	–	
Oryzias latipes	NC_019866.2	IGHD3	D	1,2,3,4,6,7,TM1	Yes	–	
Oryzias latipes	NC_019866.2	IGHM4	M	1,2,3,4,TM1	Yes	–	
Oryzias latipes	NC_019866.2	IGHD4	D	2,7,TM1	**No**	–	CD1 & CD3-6 missing (not in sequence)
Oryzias latipes	NC_019866.2	IGHM5	M	1,2,3,4,TM1	Yes	–	
Oryzias latipes	NC_019866.2	IGHD5	D	1,2,3,4,6,7,TM1	Yes	–	
Oryzias latipes	NC_019866.2	IGHM6	M	1,2,3,4,TM1	Yes	–	
Oryzias latipes	NC_019866.2	IGHD6	D	1,2,3,4,6,7,TM1	Yes	–	
Oryzias latipes	NC_019866.2	IGHD7	D	1,2,3,6	**No**	–	CD4, CD5, CD7 and TM1 missing (not in sequence)

[1] Excluding TM2 and secretory exons.

Table S28: *IGH* constant regions in cyprinidontiform fish, part 3
